# Improving the reliability, quality, and maintainability of bioinformatics pipelines with nf-test

**DOI:** 10.1093/gigascience/giaf130

**Published:** 2025-10-22

**Authors:** Lukas Forer, Sebastian Schönherr

**Affiliations:** Institute of Genetic Epidemiology, Medical University of Innsbruck, Innsbruck 6020, Austria; Institute of Genetic Epidemiology, Medical University of Innsbruck, Innsbruck 6020, Austria

**Keywords:** Nextflow, pipeline testing, test automation

## Abstract

**Background:**

The workflow management system Nextflow, together with the nf-core community, has established an essential ecosystem in bioinformatics. However, ensuring the correctness and reliability of large and complex Nextflow pipelines remains challenging due to the lack of a unified, automated unit-testing framework.

**Results:**

To address this gap, we present nf-test, a modular testing framework for bioinformatics workflows. It enables users to test process blocks, workflow patterns, and entire pipelines in isolation while validating their outputs. Built with a syntax similar to Nextflow DSL2, nf-test offers unique features such as snapshot testing and smart testing, which optimize resource usage by testing only modified modules. We demonstrate across multiple pipelines that these features minimize development time, reduce test execution time by up to 80%, and enhance software quality by identifying bugs and issues early in the development process.

**Conclusions:**

Already adopted by numerous pipelines, nf-test significantly improves the robustness, maintainability, and reliability of bioinformatics pipelines.

## Introduction

The large volumes of biological data generated across genomics, proteomics, or metabolomics have transformed bioinformatics and computational biology into big data sciences [1]. These disciplines require not only the processing of massive datasets but also the application of complex workflows to analyze, filter, and transform data to uncover complex biological relationships. Nextflow [2] has emerged as a powerful and flexible platform for building scalable and reproducible computational pipelines. Together with nf-core [3], a community-driven initiative dedicated to developing and maintaining best-practice pipelines, Nextflow has become a rich ecosystem for pipeline development [4]. However, as pipeline complexity grows, as demonstrated by several nf-core pipelines, ensuring their correctness and reliability becomes a critical challenge, especially when incorporating new features without disrupting existing functionality.

Automated testing is the process of evaluating and verifying that a software product performs as intended [5]. It is essential in scientific pipeline development to confirm functionality and ensure accurate data processing and analysis [6, 7]. The process involves defining test objectives, selecting test datasets, creating test cases, executing the pipeline with these cases, and verifying the test results [8]. Many bioinformatics pipelines face the so-called oracle problem, as they often process large input and output datasets while implementing complex algorithms without a clear gold standard [9]. This complexity makes writing test cases difficult and time-consuming. Consequently, most bioinformatics software lacks established software development quality standards [10]. An effective and comprehensive testing strategy for a pipeline’s functionality should cover multiple levels of testing, including unit testing, integration testing, and end-to-end testing. Although testing is a critical aspect of scientific software development [11], it remains underused [12]. Consequently, Nextflow pipeline maintenance demands substantial time and effort to ensure that outputs remain consistent with prior versions and scientifically valid when benchmarked against a ground truth.

Despite efforts by existing solutions to automate end-to-end testing of Nextflow pipelines [13], as well as a Python-based workflow developed by nf-core [3], a unified and robust unit-style testing framework tailored to large and complex Nextflow pipelines is still lacking. This limits the efficient and automated validation of their functionality, making it difficult for users to ensure workflow accuracy and potentially leading to errors or artifacts in data analysis and interpretation. This issue becomes even more critical when considering the clinical utility of such pipelines. Furthermore, long execution times prevent developers from rerunning tests promptly, thereby reducing productivity.

Here, we present nf-test, a testing framework designed to address these challenges within the context of Nextflow pipelines. nf-test provides a domain-specific language with a syntax similar to Nextflow DSL2 to describe the expected behavior and output data of a process or workflow. It introduces a modular approach that enables developers to isolate and validate individual process blocks, workflow patterns, and even entire pipelines. This modularity not only simplifies debugging but also promotes iterative development and code reuse. Additionally, nf-test incorporates snapshot testing and several optimization strategies to make testing data-intensive pipelines more efficient. These features help pipeline developers catch issues early in the development cycle, enabling a robust and agile development process that results in more reliable pipelines. Serving as the new standard testing framework for nf-core [3], nf-test has become an essential tool for pipeline developers. It is freely available, with extensive documentation provided on the website [14].

## Material and Methods

### Design and implementation

nf-test is implemented in Java as a command-line program, shares the same requirements as Nextflow, and is compatible with Linux and macOS. We adapted well-established testing concepts from software and web development for use in Nextflow pipeline testing. nf-test is built on a modular architecture and utilizes a plugin system, enabling effortless extension with new output formats, assertions, and optimization strategies. The software offers a wide range of project-specific configuration options and can be easily installed on continuous integration (CI) platforms using the provided installation script. Instructions, user guides, and examples are available at [14]. All code is open-source and freely available under the MIT license.

### Unit, integration, and end-to-end testing

Within the context of a Nextflow pipeline, unit testing involves evaluating a single process, workflow, or function in isolation. We developed a domain-specific language (DSL) based on Groovy that provides methods and keywords to describe the expected behavior of any Nextflow unit. A project typically comprises multiple test suites, with 1 test suite per test subject (e.g., process, workflow, or pipeline). Each test suite contains 1 or more test cases that specify the expected behavior of the test subject. A test case is defined using the “test” keyword, followed by 2 distinct blocks: (i) the “when” block, which sets the input parameters of the test subject, and (ii) the “then” block, which defines the expected output channels when the test subject is executed with the input parameters from the “when” block. Typically, the “then” block primarily contains assertions to verify assumptions, such as the content of an output channel or files. Several built-in functions simplify writing assertions and testing Nextflow channels. Additionally, the “then” block supports any Groovy script and allows the import of third-party Java libraries.

**Figure 1:**
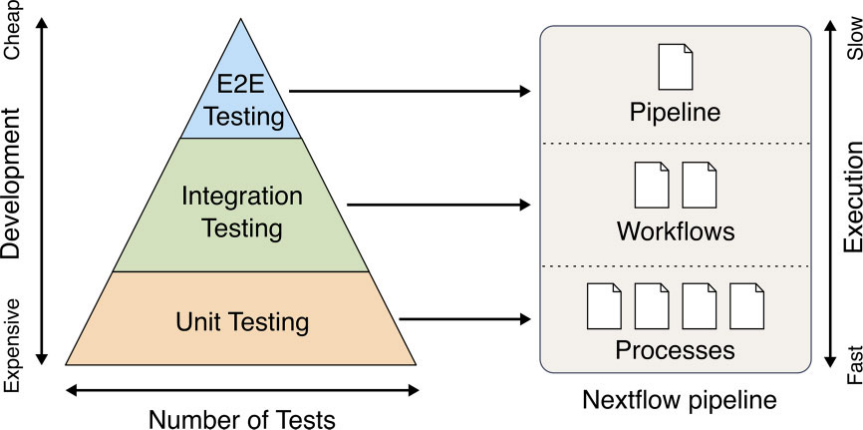
Overview of different test strategies provided by nf-test. A comprehensive and efficient test strategy for a Nextflow project includes unit, integration, and end-to-end testing. Unit tests are inexpensive to execute but require more effort to develop. In contrast, integration and end-to-end tests are easier to write but more costly to run, highlighting the trade-off between development effort and execution time across testing levels.

This modularity also enables writing integration tests for subworkflows, ensuring that individual processes interact as expected. Similarly, end-to-end tests are written in the same way, with the “when” block defining the user-provided input parameters and the “then” block verifying the expected output files. Consequently, testing is conducted consistently using the same syntax and concepts throughout the entire Nextflow project. Together, these different levels of testing provide a comprehensive and effective strategy for validating pipeline functionality, ensuring that all components work together as intended (see Fig. 1).

### Test case execution

**Figure 2:**
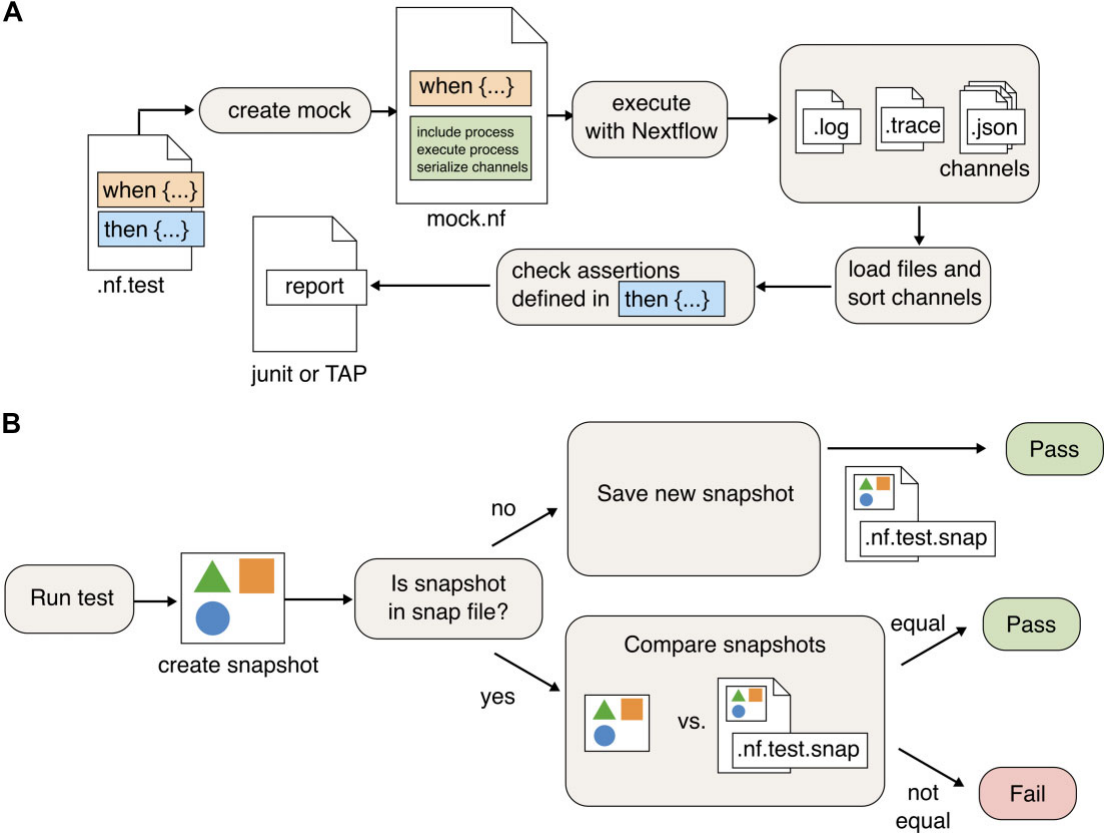
Architecture of the implemented test framework. (A) nf-test generates Nextflow scripts for tests: (i) initializes Nextflow unit with “when” block parameters, (ii) executes the unit and serializes output channels, and (iii) parses channel content and evaluates “then” block assertions for output validation. Test results are aggregated and reported in multiple formats. (B) nf-test runs a test, as well as creates and compares output objects against reference snapshot files stored with the tests. A test fails if the snapshots do not match, indicating either unexpected changes or the need to update the reference snapshot to reflect new outputs.

One or more DSL files serve as inputs for nf-test, which automatically generates the entire suite of tests. For each test, the runner automatically creates a Nextflow driver script that (i) initializes the Nextflow unit with parameters defined in the “when” block, (ii) executes the unit, and (iii) serializes all output channels. The runner then parses the output channels and evaluates the assertions defined in the “then” block to verify whether the output matches the expected behavior. Since processes are executed in parallel, Nextflow channels emit output values in a random order. nf-test ensures deterministic assertions by automatically sorting channel tuples. Finally, test results are aggregated and reported in multiple formats (e.g., JUnit, XML, TAP [15], or CSV), enabling processing by third-party reporting tools (see Fig. 2A).

### Dependency graph and smart testing

**Figure 3:**
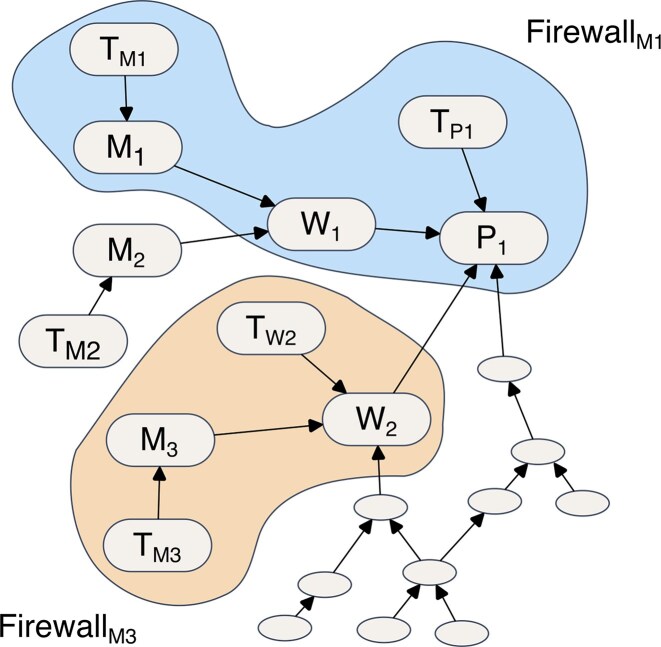
Example of a dependency graph and firewalls. The figure illustrates the dependencies of tests (*T_x_*), modules/processes (*M_x_*), workflows (*W_x_*), and pipelines (*P_x_*) within a Nextflow project. Changes to module *M*_1_ will only affect test cases inside firewall *M*_1_. Firewall *M*_3_ is more compact because workflow *W*_2_ contains a test case ensuring the integrity of *M*_3_. This approach avoids running expensive end-to-end tests for *P*_1_.

nf-test constructs a dependency graph that captures the dependencies among all modules, workflows, and test suites within a given project. In this graph, nodes represent individual modules, workflows, and test suites, while edges represent their dependencies. To build the graph, the algorithm traverses the entire project directory, identifies connections between different components (such as by parsing “include” statements), and maps them onto the graph structure. Figure 3 illustrates this concept: nodes represent processes (*M*_1_, *M*_2_, and *M*_3_), workflows (*W*_1_ and *W*_2_), and pipelines (*P*_1_), while edges denote their dependencies (e.g., *W*_1_ depends on processes *M*_1_ and *M*_2_). Test suites are linked to the components they validate (e.g., *T_M_*_1_ is the test suite for *M*_1_). Since a test suite may also depend on data or configuration files, nf-test allows users to (i) define files that always trigger a full retest (e.g., Dockerfile) and (ii) specify test assets that are automatically added to the dependency graph (e.g., input data). This directed graph provides insights into the pipeline’s architecture, supporting a comprehensive understanding of its dependencies and interactions.

Building on the dependency graph, nf-test introduces a strategy called smart testing to optimize test execution time by minimizing the number of tests run. The core idea is to use the dependency information to identify a minimal set of tests sufficient to detect regressions in modified processes or workflows. This improves efficiency, since only relevant test suites are executed. Inspired by a concept proposed by Leung and White [16, 17], nf-test implements a *firewall strategy*. When a module changes, nf-test identifies all affected nodes and constructs a firewall around them, containing only the necessary tests. Only these tests need to be retested. For example, in Fig. 3, *Firewall_M_*_1_ includes all nodes marked for retesting when module *M*_1_ is modified. Tests associated with *M*_1_ are added first, followed by the integration tests for *W*_1_, which depends on *M*_1_. Since *W*_1_ has no direct tests, we trace its dependents, in this case, *P*_1_. Because *P*_1_ has a test case, it is added to the firewall and reused to indirectly validate *W*_1_. By contrast, if *M*_3_ is modified, the firewall is smaller, as *W*_2_ has its own test case and *T_P_*_1_ does not need to be included. This approach enables fine-grained control over the firewall, allowing expensive end-to-end tests to be excluded where appropriate, while maintaining accuracy and reducing testing costs.

### Git support and testing status

As most Nextflow pipelines use version control, nf-test also integrates with Git [18] to automatically detect changes in the working tree. This enables test execution on differences between commits or branches, ensuring continuous validation of pipeline modifications across commits and releases.

The testing status is determined by analyzing the dependency graph to identify which components are directly or indirectly verified by at least 1 test case. This provides an overview of which parts of the workflow are exercised by tests, without implying complete or line-level testing. Additionally, the testing status can be computed for a firewall to provide a safety-related status metric.

### Parallelization with test list sharding

Parallelization is achieved through test list sharding [19], which splits the test suite into smaller subsets and distributes them across multiple parallel execution environments or machines. Discovered tests are sorted by type and filename to ensure a deterministic order across machines. To optimize test distribution across n machines, nf-test provides 2 strategies: (i) a simple chunking algorithm that splits the test list into *n* chunks and (ii) a round-robin approach for equitable allocation (“–*shard-strategy round-robin*”). For example, splitting a suite into 3 shards can be done by running one of the following commands on each machine: “*nf-test –shard 1/3*,” “*nf-test –shard 2/3*,” and “*nf-test –shard 3/3*.”

### Snapshot testing

Snapshot testing is a technique commonly utilized in web development [20] and has been adapted in nf-test for Nextflow pipelines. Its naming and parameters are inspired by Jest [21]. nf-test captures a snapshot of output channels or other provided objects and compares them to reference snapshot files stored alongside the tests. Each snapshot file is a JSON file containing a serialized version of its content. When a file is added to a snapshot, its MD5 hash is stored instead of the file content. Additionally, nf-test can save the MD5 hash of an entire snapshot, enabling a compressed representation of large or complex content.

Because snapshot files are simple text files, they can be checked into version control systems and support human-readable diffs. During each test run, nf-test compares the actual snapshot with the reference snapshot. If the 2 snapshots differ, the test fails (see Fig. 2B). If the change is unexpected, the user should address the bug detected by the test. Otherwise, the reference snapshot must be updated to reflect the new output of a process, workflow, pipeline, or function. In this case, the user runs nf-test with the “*–update-snapshot*” option.

All snapshot files are automatically included in the dependency graph, ensuring that tests are triggered whenever a snapshot changes. In addition, nf-test provides a CI mode (*–ci*), which prevents automatic snapshot updates and enforces test failures when discrepancies occur.

### Extensions for bioinformatics

nf-test provides a plugin system for reusing code snippets, saving development time, simplifying test code, and enhancing maintainability. The plugin system, built on Groovy, is well documented to encourage users to create and share their own extensions. For example, in most bioinformatics file formats, file determinism is not always guaranteed, as timestamps or input filenames can prevent files from being byte-identical. In such cases, MD5 hashs cannot be used for verification, and implementing tests to validate the dynamic output can be time-consuming. To address this, nf-test provides plugins with built-in methods to extract and validate information from well-known bioinformatics file formats. Currently, nf-test includes plugins for VCF, BAM, FASTA, FASTQ, and CSV files (see [22]). For example, the nft-vcf plugin allows users to access file summaries, metadata, individual variants, and other key attributes, enabling flexible and automated testing. These plugins simplify writing nf-test assertions and make testing more convenient and powerful for end users.

### Evaluation and validation

We evaluated nf-test using 3 publicly available Nextflow pipelines to demonstrate its effectiveness in pipeline testing. While these examples focus on nf-core pipelines, nf-test is a general-purpose framework that can also be applied to other Nextflow-based projects outside the nf-core ecosystem. For instance, nf-test has been used with the publicly available genotype imputation pipeline employed by popular imputation servers [23]. We first assessed the effect of smart testing on optimizing test execution times and parallelization (see Results section “Optimizing Testing Efficiency and Performance”). This was done by simulating 4 specific changes through manual file modifications in the nf-core/fetchngs (version 1.12.0, [24]) pipeline. We ran nf-test with the “*–related-tests*” option, which executes only tests relevant to the specified files. We also evaluated nf-core/modules (Commit ca199cf, [25]), a repository whose modules are already covered by nf-test tests, by running nf-test on the last 500 commits, using the “*–changed-since HEAD^*” flag to capture changes between consecutive versions. Second, to evaluate parallelization, we implemented 39 test cases and set up CI using GitHub Actions for the nf-gwas pipeline (version 1.05, [18]). We tested it by employing test-list sharding across 5 machines with the option “*–shard i/5*,” comparing both default and round-robin strategies for test case distribution. Speedup was measured as the ratio of execution times with and without test-list sharding. All analyses were conducted using nf-test 0.9.0, and results were visualized using R 4.3.3 and ggplot2 3.5.0.

## Results

### Optimizing testing efficiency and performance

#### Resource saving through smart testing

First, we evaluated smart testing and its impact on execution time using the nf-core/fetchngs pipeline. This pipeline includes 50 test cases for 17 components. The dependency graph illustrates the connections and dependencies between modules, workflows, and test cases. Because each component includes at least 1 test case, the pipeline achieves 100% coverage (see Supplementary Fig. S1). The total execution time for all tests is 1,122 seconds. As hypothesized, we observed that pipeline end-to-end tests exhibit the slowest performance (see Table 1). We simulated various modifications to assess the impact of changes, varying the number of modified files and the type of change: (i) changes to the logic of the module itself and (ii) changes to the module interface (e.g., adding a new input channel). Results show that smart testing saves between 46% and 80% of execution time by minimizing the number of executed test cases (see Table 2). We reviewed the results from a full run of the entire test suite and confirmed that all changes were detected by our approach.

**Table 1:** Number of test cases in the nf-core/fetchngs pipeline (version 1.12.0). Each of the 17 components has at least 1 test case, for a total of 50 test cases. The total execution time is 1,122.1 seconds.

			Execution time (s)	
	Test suites	Test cases	Mean	Total
Functions	3	14	2.6	36.5
Pipelines	1	1	512.1	512.1
Modules/processes	10	13	11.2	145.7
Workflows	15	22	19.4	427.8
Total	29	50	—	1,122.1

**Table 2:** Time and resource saving for different modifications of the nf-core/fetchngs. We simulated several typical modifications and measured the execution time using nf-test’s optimization strategy. Time savings are calculated based on the execution time of a full run (1,122.1 seconds).

		Execution time		
Modification	Executed test cases	Mean (s)	Total (s)	Saving
changed module sra_to_samplesheet	10	22.3	222.5	80.2%
changed modules sra_to_samplesheet multiqc_mappings_config	11	52.7	579.4	48.4%
update of a nf-core module: utils_nfcore_pipeline	23	14.4	332.1	70.4%
changed main workflow main.nf	1	596.3	596.3	46.8%

**Figure 4:**
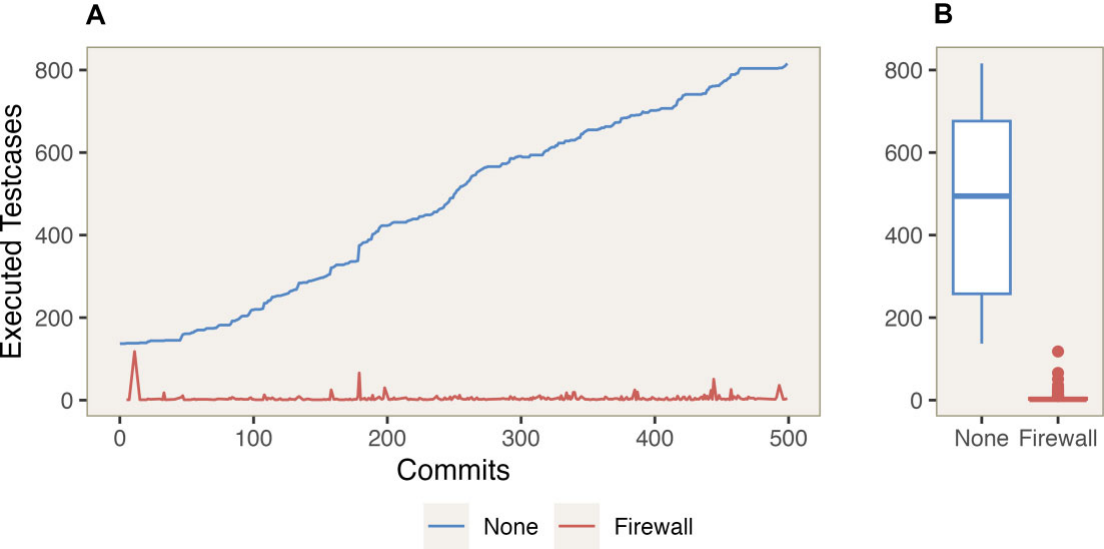
Last 500 commits of the nf-core/modules projects between 26 October 2023 and 23 February 2024. (A) The blue line represents the number of test cases that would be executed without any optimization strategy. The red line depicts the number of executed test cases when using the implemented firewall strategy. (B) Boxplot of the number of executed test cases per commit.

Second, we analyzed the last 500 commits in the nf-core/modules project, spanning from 26 October 2023 to 23 February 2024. At the time of writing, nf-core/modules contain 1,150 modules, 56 workflows, and more than 800 test cases implemented by the community. As expected, most commits and pull requests affect only a single module. In such cases, time savings are significantly higher, as only the relevant unit tests and potential workflow integration tests need to be executed. nf-test accurately identified the specific test cases and integration tests required for the committed changes (see Fig. 4A). Approximately 30% of the commits required executing more than 25 test cases, with most of these commits involving refactoring or restructuring tasks (see Fig. 4B). The total number of executed test cases was reduced from 238,205 to 1,600. nf-test parsed and analyzed 1,560 unique files in under 1 second to construct the dependency graph.

#### Execution time reduction through parallelization

We evaluated the efficiency and speedup of parallelization using nf-gwas [26], a pipeline for genome-wide association studies, which includes multiple long-running end-to-end tests (see Supplementary Fig. S2 for the dependency graph). Initially, executing all 49 test cases on a single machine required 1,718 seconds. Execution time was reduced to 487 seconds by distributing the workload across 5 machines, achieving a speedup of 3.5. The default strategy distributes tests by name, which can result in unbalanced execution times, especially when outliers are present (see Table 3). Using a round-robin approach further reduced execution time to 333 seconds, resulting in a speedup of 5.2.

**Table 3:** Time and speedup for different sharding strategies of the nf-gwas pipeline

Strategy	Shards	Median (s)	Total time (s)	Speedup
None	5	368 ±164	487	3.52
Round-robin	5	314 ± 10	333	5.15

#### Code reduction through snapshot testing

We analyzed how snapshot testing in nf-test can improve quality and maintainability by reducing the effort required to write. Writing manual assertions for each output item can be time-consuming and error-prone, especially in pipelines with large numbers of outputs. For example, the nf-gwas pipeline generates 5 output files for a single phenotype. Writing a simple regression test requires creating an assertion for each file to verify its existence and another to ensure its content matches expectations. This manual process entails generating an MD5 hash for each file and using it in the corresponding assert block, resulting in 15 lines of code for 3 phenotypes. Additionally, the test case must be updated whenever the pipeline generates additional output files, requiring manual synchronization. Snapshot testing streamlines this process by replacing 15 lines of code with a single line. nf-test automatically creates the MD5 hash for each file on the first run and provides commands to update the reference snapshot.

We adapted the nf-gwas pipeline and simulated various software updates for REGENIE [27], the underlying software: (i) changes in the output file format (renaming the column “LOG10P” to “PVALUE”), (ii) a bug in association detection (where 6 variants are no longer genome-wide significant), and (iii) modifications to default parameters. Change (i) breaks the pipeline and is easy to detect without test cases by running the pipeline on test data. However, changes (ii) and (iii) produce incorrect output results without breaking the pipeline. While these scenarios are particularly challenging for a pipeline developer, parameter or result changes can also outputs that are still valid and, in some cases, even more biologically meaningful. The implemented test cases detected all of these issues. For example, the unit test for REGENIE, which checks whether 116 variants are genome-wide significant, fails because only 110 variants were found. Similarly, changes in default parameters (e.g., a more restricted MAF filter) produce a different number of resulting variants, causing the test to fail.

### Comparison to other frameworks

nf-test is a flexible testing framework for Nextflow pipelines, going beyond existing solutions such as NFTest and pytest-workflow (see Table 4). Unlike these systems, nf-test integrates unit, snapshot, and end-to-end testing into a single extendable DSL, while also supporting advanced features like optimized test strategies, dependency analysis, coverage reporting, and parallelized execution. It introduces bioinformatics-aware assertions (e.g., for VCF, BAM, or FASTA files), modular and portable test design, and multiple output formats (JUnit, XML, TAP, CSV), making it highly suitable for large-scale scientific workflows. In contrast, NFTest and pytest-workflow provide more limited functionality, requiring a manual setup for unit or snapshot testing and lacking support for advanced optimizations, dependency tracking, or domain-specific assertions. nf-test combines flexibility, reproducibility, and portability in a way that existing tools do not. nf-test has established itself as the tool of choice within the Nextflow and nf-core communities, which provide best-practice pipelines for a wide range of use cases.

**Table 4:** Comparison of nf-test with similar approaches

	nf-test	NFTest	pytest-workflow
**End-to-end testing**	Yes	Yes	Yes
**Unit testing**	Yes	Manual writing Nextflow script	Manual writing Nextflow script
**Snapshot testing**	Yes	Manual preparation of expected output files	Manual preparation of expected output files
**Optimized test strategies**	Yes	No	No
**Dependency analysis**	Yes	No	No
**Coverage reporting**	Yes	No	No
**Tags**	Yes	No	Yes
**Output formats**	Junit, xml, TAP, and csv	No	Junit, html
**Code generation**	config file and for each unit	config file	Using nf-core tools
**Custom assertions**	Yes	Through external third-party scripts	No
**Bioinformatics support**	Yes (e.g., vcf, fasta, …)	No	No
**Parallelization**	Sharding	No	No
**Modularity and portability**	Each unit has its own test files that can be transferred	One test file for whole project	Multiple files
**Writing test cases**	Extendable DSL	YAML with predefined structure	YAML with predefined structure
**License**	MIT	GPL-2.0	AGPL-3.0
**Website**	https://www.nf-test.com	https://github.com/uclahs-cds/tool-NFTest	https://pytest-workflow.readthedocs.io

## Discussion

Since its first release in October 2021, nf-test has been integrated into dozens of pipelines and downloaded over 200,000 times. As the new standard testing framework in nf-core for both pipelines and provided modules, it shows a high level of acceptance, reflecting the community’s recognition of the importance of testing pipelines.

Various efforts have previously addressed Nextflow pipeline testing, including NFTest [13] and the nf-core framework [3], which utilize pytest-workflow [28]. However, these solutions rely on YAML files with predefined assertions, are limited to end-to-end testing, depend on external scripts to validate output files, and do not support optimization strategies such as parallelization and smart testing. nf-test addresses these limitations by providing a dedicated testing framework that simplifies writing, executing, and analyzing tests for Nextflow pipelines. It offers a DSL that follows the naming conventions and philosophy of Nextflow DSL 2 and enables writing complex assertions, which are often required to validate the extensive outputs of bioinformatics analysis. Because the DSL is based on Groovy, users can extend it and leverage the rich ecosystem of Java/JVM libraries in bioinformatics. Additionally, sharing domain-specific assertions via plugins facilitates collaboration among users.

We implemented a unit testing approach in which all components of a Nextflow pipeline can be tested without manually writing additional Nextflow workflows to execute a subprocess with test data. This modularity also facilitates writing integration tests for subworkflows, ensuring that processes interact as expected. Testing is conducted consistently across a project, allowing side effects to be detected early. The modular design simplifies debugging, encourages iterative development, and promotes code reuse. Each module can be individually tested and seamlessly integrated into the final pipeline or workflow composition. Integration tests are particularly important when using large module libraries, such as those provided by nf-core/modules, to ensure that third-party updates do not break pipelines.

In pipelines with extensive outputs, manually writing assertions for each output item can be complex. To address this, nf-test introduces snapshot testing. Instead of specifying individual assertions for each output, snapshots capture the state of the output channels or folders, including file names and hash values. These snapshots are automatically generated during the first run, and nf-test compares the current output with the reference snapshot in subsequent runs. This approach streamlines regression testing, ensures reproducibility, and helps catch regressions early in development. Snapshot testing in nf-test is particularly useful in 2 scenarios: first, it supports pipeline and module refactoring, where outputs are expected to remain unchanged, ensuring that modifications to the Nextflow logic do not alter the results. Second, it aids version management, as test failures can indicate changes in underlying tools or dependencies, signaling that a new version or review may be required. Outside of these cases, traditional testing approaches are generally more appropriate. nf-test therefore also provides mechanisms for assertion-based tests, allowing verification of file or channel contents, tool execution checks to confirm expected behavior for given inputs, and error-handling tests that ensure robust failure responses. Together, these testing strategies provide a balanced framework that combines the change-detection benefits of snapshot testing with the precision of conventional verification methods. Evaluation of the nf-gwas pipeline demonstrated that nf-test and snapshot testing improve code quality and maintainability while reducing the effort required to write manual assertions.

Regression testing involves retesting pipelines after any code modification. Given that bioinformatics pipelines process large input data and employ complex algorithms, full regression tests can take hours. To save effort and time, nf-test only reruns tests affected by a modification. Using the nf-core/modules project as an example, we showed that most changes and commits in such large projects affect only a specific set of modules and tests. Therefore, nf-test implements strategies to identify the minimal set of tests that must be rerun. This results in resource savings and faster development cycles, up to an 80% reduction in execution time.

When pipelines contain large numbers of tests, test-list sharding can split tests across multiple machines. Experimental results showed performance gains of up to 80% using 5 machines. However, there is no guarantee of an optimal or fair split among resources as the splitting decision is not influenced by data from previous runs. The implemented round-robin strategy simply attempts to distribute the workload evenly. Nonetheless, the setup remains easy since no shared database, queuing system, or orchestration instance is needed. Combined with the integration of Git, nf-test enables the setup of test-driven development and CI for Nextflow pipelines.

The implemented dependency analysis provides an overview of pipeline coverage and quantifies the testing effort. The current implementation has limitations, as it only indicates whether at least 1 test case exists per unit, without reflecting whether all instructions or branches are covered. Future work aims to extend this approach and to include metrics that reflect the complexity of change sets. Additionally, nf-test currently depends on local environments, which can limit adaptability to certain infrastructures. For example, running test cases across different cloud providers is currently limited and will be addressed in future versions.

## Availability of Source Code and Requirements

Project name: nf-test

Project homepage: https://github.com/askimed/nf-test

Operating system(s): Platform independent

Programming language: Java

License: MIT

RRID:SCR_026663

## Additional Files

**Supplementary Fig. S1**. Dependency graph of nf-core/fetchngs. Green rectangles represent Nextflow files, blue rectangles indicate test cases for these files, and white rounded rectangles denote snapshots.

**Supplementary Fig. S2**. Green rectangles represent Nextflow files that have at least 1 test case, while red indicates those without a test case. Blue rectangles indicate test cases for these files, and white rounded rectangles denote snapshots.

giaf130_Supplemental_Material

giaf130_Authors_Response_To_Reviewer_Comments_Original_Submission

giaf130_GIGA-D-25-00116_Original_Submission

giaf130_GIGA-D-25-00116_Revision_1

giaf130_Reviewer_1_Report_Original_Submission Jose Espinosa-Carrasco -- 5/19/2025

giaf130_Reviewer_2_Report_Original_Submission Katalin Ferenc -- 7/1/2025

## Abbreviations

CI: continuous integration; DSL: domain-specific language.

## Data Availability

nf-test and its documentation are available at nf-test [29] and Bioconda [30]. Associated plugins are available at [31].
