# Supplementary material for: Improving the reliability, quality, and maintainability of bioinformatics pipelines with nf-test
Source: Gigascience. 2025 Oct 22;14:giaf130. doi: 10.1093/gigascience/giaf130 (PMC12616847; doi:10.1093/gigascience/giaf130)

Supplemental Material

Improving the Reliability, Quality and Maintainability of Bioinformatics Pipelines with nf-test

Lukas Forer[1*] and Sebastian Schönherr[1]

1. Institute of Genetic Epidemiology, Medical University of Innsbruck, Innsbruck, Austria

* Corresponding Author:

Lukas Forer, PhD.

lukas.forer@i-med.ac.at

Institute of Genetic Epidemiology

Medical University of Innsbruck

Schöpfstrasse 3

6020 Innsbruck, Austria

Figures

Figure S1: Dependency graph of nf-core/fetchngs. Green rectangles represent Nextflow files, blue rectangles indicate test cases for these files and white rounded rectangles denote snapshots.

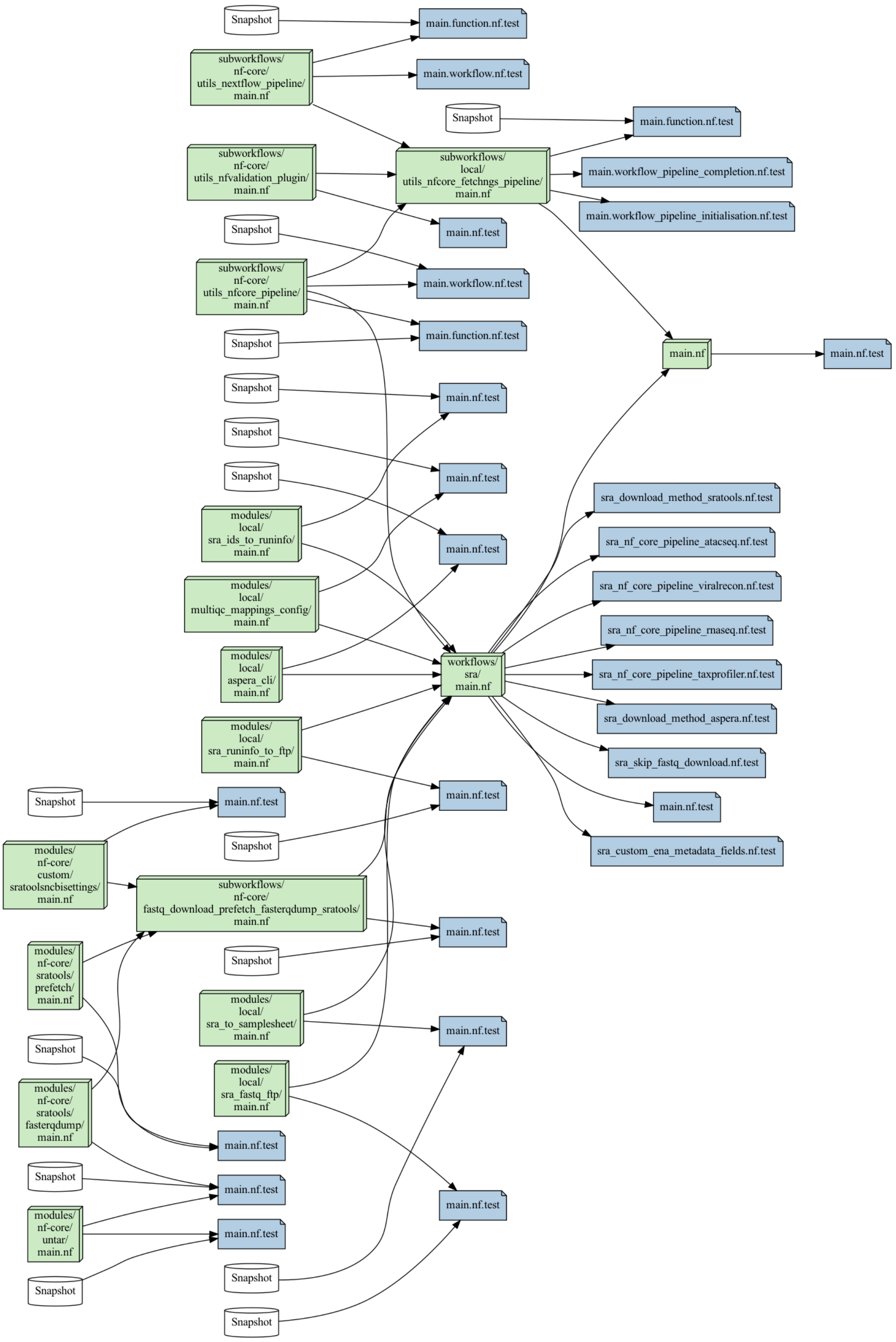

Figure S2: Dependency graph of nf-gwas pipeline. Green rectangles represent Nextflow files that have at least one test case, while red indicates those without a test case. Blue rectangles indicate test cases for these files and white rounded rectangles denote snapshots.

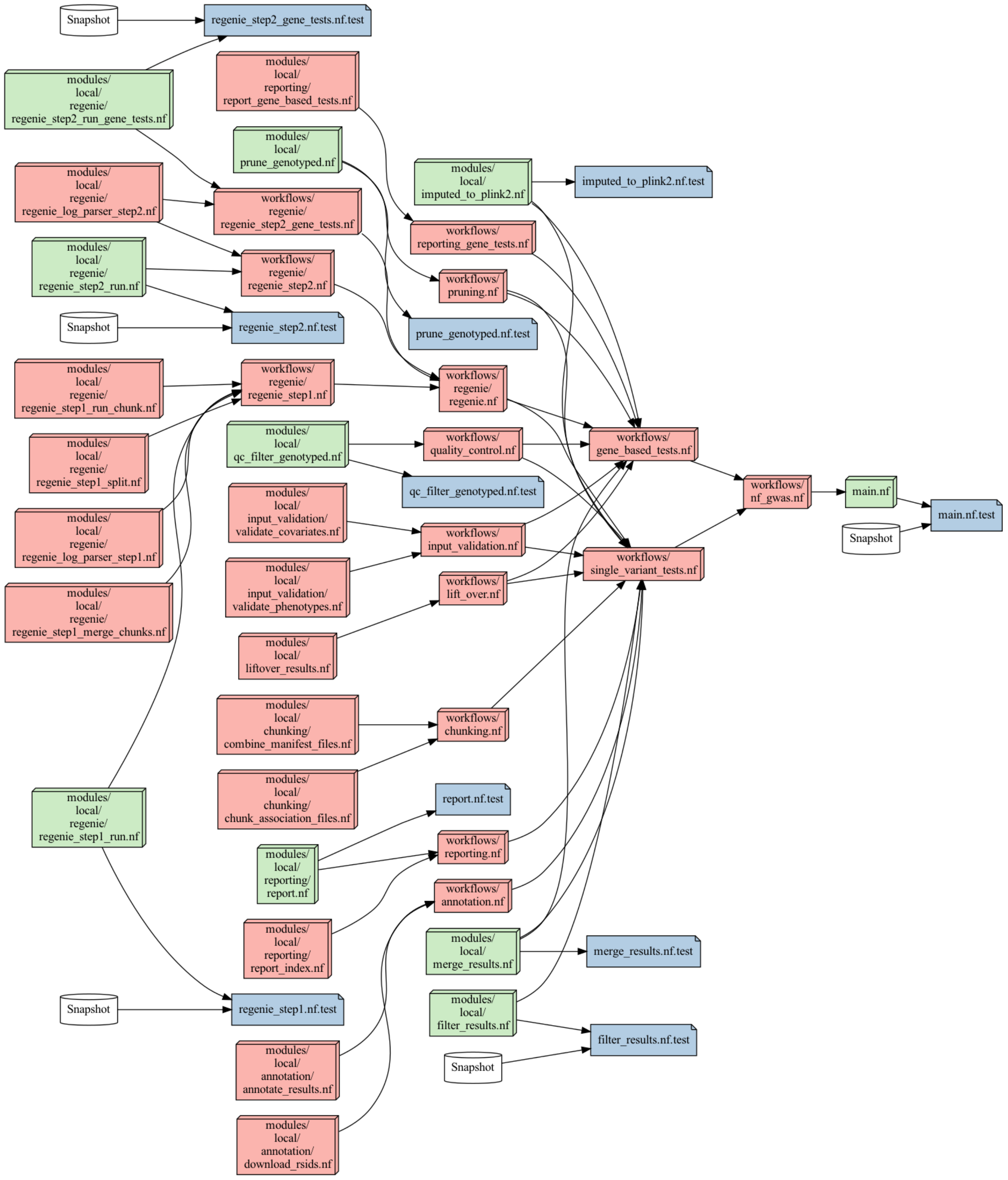

Supplement: giaf130_Supplemental_Material [file giaf130_supplemental_material.pdf]
