# Supplementary material for: Improving the reliability, quality, and maintainability of bioinformatics pipelines with nf-test
Source: Gigascience. 2025 Oct 22;14:giaf130. doi: 10.1093/gigascience/giaf130 (PMC12616847; doi:10.1093/gigascience/giaf130)
Supplement: giaf130_GIGA-D-25-00116_Original_Submission [file giaf130_giga-d-25-00116_original_submission.pdf]

GigaScience

Improving the Reliability, Quality and Maintainability of Bioinformatics Pipelines with nf-test

--Manuscript Draft--

**Manuscript Number:**	GIGA-D-25-00116
**Full Title:**	Improving the Reliability, Quality and Maintainability of Bioinformatics Pipelines with nf-test
**Article Type:**	Research
**Funding Information:**	
**Abstract:**	The workflow management system Nextflow builds together with the nf-core community an essential ecosystem in bioinformatics. However, ensuring the correctness and reliability of large and complex Nextflow pipelines is challenging due to the lack of a unified, automated unit-testing framework. To fill this gap, we present nf-test, a modular testing framework for bioinformatics workflows. It enables bioinformaticians to test process blocks, workflow patterns, and entire pipelines in isolation while validating their outputs. Build on a similar syntax as Nextflow DSL2, nf-test offers unique features such as snapshot testing and smart testing, which optimizes resource usage by testing only modified modules. We demonstrate on different pipelines that these features minimize the development time, reduce test execution time by up to 80% and enhance software quality by identifying bugs and issues early in the development process. Already used by numerous pipelines, nf-test significantly improves the robustness, maintenance and reliability of bioinformatics pipelines.
**Corresponding Author:**	Lukas Forer<br>Medical University of Innsbruck: Medizinische Universitat Innsbruck<br>Innsbruck, AUSTRIA
**Corresponding Author Secondary Information:**	
**Corresponding Author's Institution:**	Medical University of Innsbruck: Medizinische Universitat Innsbruck
**Corresponding Author's Secondary Institution:**	
**First Author:**	Lukas Forer
**First Author Secondary Information:**	
**Order of Authors:**	Lukas Forer
	Sebastian Schönherr
**Order of Authors Secondary Information:**	
**Additional Information:**	
**Question**	**Response**
Are you submitting this manuscript to a special series or article collection?	No
**Experimental design and statistics**<br><br>Full details of the experimental design and statistical methods used should be given in the Methods section, as detailed in our Minimum Standards Reporting Checklist. Information essential to interpreting the data presented should be made available	Yes

Powered by Editorial Manager® and ProduXion Manager® from Aries Systems Corporation

in the figure legends.<br><br>Have you included all the information requested in your manuscript?	
**Resources**<br><br>A description of all resources used, including antibodies, cell lines, animals and software tools, with enough information to allow them to be uniquely identified, should be included in the Methods section. Authors are strongly encouraged to cite Research Resource Identifiers (RRIDs) for antibodies, model organisms and tools, where possible.<br><br>Have you included the information requested as detailed in our Minimum Standards Reporting Checklist?	Yes
**Availability of data and materials**<br><br>All datasets and code on which the conclusions of the paper rely must be either included in your submission or deposited in publicly available repositories (where available and ethically appropriate), referencing such data using a unique identifier in the references and in the "Availability of Data and Materials" section of your manuscript.<br><br>Have you have met the above requirement as detailed in our Minimum Standards Reporting Checklist?	Yes
GigaScience has policies and guidelines in place for the use of generative AI-writing tools such as ChatGPT. If you have used such writing tools to assist with writing the manuscript this must be declared and cited in the text. Authors should not list AI-writing tools and other AI-assisted technologies as an author or co-author and should acknowledge that they are fully responsible for text generated or refined by AI-writing	No

tools.<p><br><br>A summary of use (particularly in the introduction or among methods) needs to be included at the end of the paper, and the outputs should also be included as a supplementary file hosted in GigaDB or other open repositories. Please read our guidelines for more information. <p><br><br>By submitting to GigaScience, you are aware of the journal's AI-writing tools policy, and if you have declared use of such tools below, you have acknowledged this where appropriate in your manuscript and have made a summary of use and outputs available. <p><br><b>AI-assisted writing tools have been used in the preparation of this manuscript?	

Powered by Editorial Manager® and ProduXion Manager® from Aries Systems Corporation

Manuscript Click here to access/download;Manuscript;manuscript.docx

Improving the Reliability, Quality and Maintainability of Bioinformatics Pipelines with nf-test

Lukas Forer[1*] and Sebastian Schönherr[1]

1. Institute of Genetic Epidemiology, Medical University of Innsbruck, Innsbruck, Austria

* Corresponding Author:

Lukas Forer, PhD.

lukas.forer@i-med.ac.at

Institute of Genetic Epidemiology

Medical University of Innsbruck

Schöpfstrasse 3

6020 Innsbruck, Austria

ABSTRACT

The workflow management system Nextflow builds together with the nf-core community an essential ecosystem in bioinformatics. However, ensuring the correctness and reliability of large and complex Nextflow pipelines is challenging due to the lack of a unified, automated unit-testing framework. To fill this gap, we present nf-test**,** a modular testing framework for bioinformatics workflows. It enables bioinformaticians to test process blocks, workflow patterns, and entire pipelines in isolation while validating their outputs. Build on a similar syntax as Nextflow DSL2, nf-test offers unique features such as snapshot testing and smart testing, which optimizes resource usage by testing only modified modules. We demonstrate on different pipelines that these features minimize the development time, reduce test execution time by up to 80% and enhance software quality by identifying bugs and issues early in the development process. Already used by numerous pipelines, nf-test significantly improves the robustness, maintenance and reliability of bioinformatics pipelines.

Keywords: Nextflow, Pipeline Testing, Test Automation

INTRODUCTION

Due to the large amounts of biological data generated from various sources within genomics, proteomics, or metabolomics, the disciplines of bioinformatics and computational biology have become a big data science [1]. It involves processing large datasets as well as applying complex workflows to analyze, filter and transform them to uncover complex biological relationships. Nextflow [2] has emerged as a powerful and flexible platform for building scalable and reproducible computational pipelines in the field of bioinformatics. In conjunction with nf-core [3], a community-driven initiative dedicated to developing and maintaining best practices pipelines, a rich ecosystem has evolved [4]. However, as pipeline complexity grows, ensuring their correctness and reliability becomes a critical challenge, especially when incorporating new features without disrupting existing ones. While testing is a critical aspect of scientific software development [5], it remains underused in scientific software [6]. Thus, maintenance requires substantial time and effort to manually verify that the pipeline continues to produce scientifically valid results.

Automated testing is the process of evaluating and verifying that a software product does what it is supposed to do [7]. It is essential in scientific pipeline development to confirm the pipeline's functionality and to ensure accurate data processing and analysis [8, 9]. It further involves defining test objectives, selecting test datasets, creating test cases, executing the pipeline with these test cases and verifying the test results [10]. Many bioinformatics pipelines suffer from the so-called oracle problem, as they often process large input and output datasets while implementing complex algorithms without a clear gold standard [11]. This complexity makes the process of writing test cases difficult and time-intensive. As a result, most bioinformatics software lacks established software development quality standards [12]. A comprehensive and effective testing strategy for the pipeline's functionality needs to cover different test levels and is divided into unit testing, integration testing and end-to-end testing. Unit testing plays a crucial role in software testing by verifying the correctness of individual units or components

of code. Integration testing is important to ensure that different processes within a larger workflow interact as expected. Finally, end-to-end testing involves testing the entire pipeline from start to finish, as a user would run it.

Despite efforts from existing solutions to automate end-to-end testing of Nextflow pipelines [13], a unified and robust unit-style testing framework specific to large and complex Nextflow pipelines is still lacking. This limits the efficient and automated validation of their functionality, making it difficult for bioinformaticians to guarantee the accuracy of their workflows and potentially leading to errors and artifacts in data analysis and interpretation. This issue becomes even more critical when considering the clinical utility of such pipelines. Moreover, long execution times hinder developers from rerunning tests promptly, thereby limiting their productivity.

Here we present nf-test, a testing framework designed to address these challenges within the context of Nextflow pipelines. nf-test provides a domain-specific language with a syntax similar to Nextflow DSL 2 to describe the expected behavior and output data of a process or workflow. It introduces a modular approach that enables developers to isolate and validate individual process blocks, workflow patterns, and even entire pipelines. This modularity not only simplifies the debugging process but also promotes iterative development and code reuse. Moreover, we introduce snapshot testing and provide several optimization strategies to make testing data-intensive pipelines more efficient. All of this helps pipeline developers to catch issues early in the development cycle and enables a robust and agile development process that results in more reliable pipelines. Serving as the new standard testing framework for nf-core [3], nf-test emerges as an essential tool for pipeline developers in the field of bioinformatics. It is freely available, and extensive documentation is provided on the website.

MATERIAL AND METHODS

Design and Implementation

nf-test is implemented in Java as a command-line program. We adapted well-established testing concepts from software and web development to Nextflow pipeline testing. nf-test is built upon a modular architecture and utilizes a plugin system, enabling effortless extension with new output formats, assertions, and optimization strategies. All code is open-source and freely available under the MIT License.

Smart Testing

Dependency Analysis

nf-test constructs a dependency graph that outlines the dependencies among all modules, workflows, and test suites within a given project. In the graph, nodes represent individual modules, workflows and test suites, while edges represent the dependencies between them. The developed algorithm to create and discover these dependencies traverses the entire project directory, identifies connections between different components (such as by parsing “include” statements), and maps them onto the graph structure. In **Figure 1**, an illustrative example showcases this concept. The nodes represent different processes (M_1, M_2 and M_3), workflows (W_1 and W_2) as well as pipelines (P_1), and are connected by edges denoting their dependencies (for example, W_1 depends on processes M_1 and M_2). Test suites are connected to their testing subject (for instance, T_{M1} is the test suite for M_1). As a test suite could also be dependent on data or configuration files, it is possible to (a) define a list of files that always trigger a full retest (e.g. Dockerfile), and (b) define the assets of test cases that are automatically added to the dependency graph (e.g., input data). This directed graph offers insights into the underlying architecture of the pipeline, facilitating a comprehensive understanding of its dependencies and interactions.

Optimizing Test Execution

The dependency graph is used to implement a cost-effective testing strategy called smart testing. The main idea is to use the dependency information to identify a minimal set of tests required to detect potential regressions for changed processes or workflows. This improves efficiency and effectiveness in test execution since only specific test suites need to be retested. Inspired by a concept proposed by Leung and White [14, 15], we implemented a strategy based on *firewalls*. nf-test identifies all nodes affected by the modified module and constructs a firewall around the nodes, containing only tests that are affected by the change. Only these tests need to be retested. For instance, in **Figure 1**, $Firewall_{M1}$ contains all nodes that have been detected for retesting if module M_1 is changed. Tests associated with M_1 are first added to the firewall, followed by the inclusion of integration tests for W_1, because it depends on M_1. Since W_1 has no direct tests, we trace all nodes that depend on W_1, in this case, P_1. For P_1, a test case exists, which is added to the firewall and can also be reused to indirectly test *W1*. On the other hand, if M_3 is modified, the resulting firewall is smaller, since W_2 has its own test case and there is no need to add T_{P1} to the firewall. This strategy enables us to refine the firewall by potentially excluding expensive end-to-end tests while maintaining accuracy and reducing testing costs.

Test Coverage Calculation

Test coverage is calculated by analyzing the dependency graph and determining which components are directly or indirectly covered by a test. The percentage of covered components relative to the total number of components provides a measure of the test coverage, indicating how much of the functionality is validated by the tests. Additionally, coverage can be calculated for a firewall to obtain a metric of how safe or well-tested a given change set is.

Parallelization due to Test List Sharding

We implemented test list sharding to split the tests across several machines [16]. The discovered tests are sorted by type and filenames to ensure a deterministic order across different machines. To optimize test distribution across *n* machines, we provide two distinct strategies: (a) a simple chunking algorithm that splits the test list into *n* chunks and (b) a round-robin approach for equitable allocation (`` `--shard-strategy round-robin` ``). For instance, splitting a suite into three shards for distributed processing can be achieved by running one of the three commands on each machine: `` `nf-test --shard 1/3` ``, `` `nf-test --shard 2/3` ``, and `` `nf-test --shard 3/3` ``.

Snapshot Testing

Snapshot testing is a technique commonly utilized in web development [17] and has been adapted by us to apply it to Nextflow pipelines. The naming and parameters are heavily inspired by Jest (https://jestjs.io). nf-test captures a snapshot of output channels or any other provided object and subsequently compares them to reference snapshot files stored alongside the tests. The snapshot file is a JSON file that contains, for each snapshot, a serialized version of its content. When a file is added to a snapshot, its MD5 hash sum is automatically included instead of the file content itself. Additionally, it is possible to save the MD5 sum of the entire content of a snapshot, enabling the storage of a compressed version of complex and large content. Since snapshot files are simple text files, they can easily be checked into version control systems and support human-readable differences. During each test run, nf-test compares the actual snapshot with the reference snapshot. If the two snapshots do not match, the test will fail. If the change is unexpected, the user can address the bug detected by the test. Otherwise, the reference snapshot needs to be updated to the new output of a process, workflow, pipeline, or function. In this case, the user can run nf-test with the option `` `--update-snapshot` ``.

Furthermore, the associated snapshot files are automatically included in the dependency graph to ensure that test modifications are triggered whenever a snapshot changes. We implemented a special continuous-integration (CI) mode that can be activated with the `--ci` flag. When enabled, this mode prevents nf-test from updating the snapshot, causing the test to fail.

Evaluation and Validation

We evaluated nf-test with three publicly available Nextflow pipelines to demonstrate its effectiveness in pipeline testing. First, we assessed nf-core/fetchngs (Version 1.12.0, https://github.com/nf-core/fetchngs) by simulating four specific changes through manual file modifications and running nf-test with the `*--related-tests*` option. Next, we implemented 39 test cases and Continuous Integration (CI) using GitHub Actions for the nf-gwas pipeline (Version 1.05, [18]). We tested it by employing test-list sharding across five machines with the option`*--shard i/5*`, comparing both default and round-robin strategies for test case distribution. Speedup was measured as the ratio of execution times with and without sharding. Finally, we evaluated nf-core/modules (Commit ca199cf, https://github.com/nf-core/modules) by running nf-test on the last 500 commits, utilizing the `*--changed-since HEAD^*` flag to capture changes between consecutive versions. All analyses were conducted using nf-test 0.9.0 and the results were visualized using R 4.3.3 and ggplot2 3.5.0.

RESULTS

nf-test framework

nf-test is implemented as a command-line program, shares the same requirements as Nextflow, and is compatible with Linux or macOS. We adapted well-established testing concepts from software and web development to Nextflow pipeline testing. The software offers a wide range of project-specific configuration options and can be easily installed on Continuous Integration (CI) platforms using the provided installation script. Instructions, user guides, and examples are available at https://www.nf-test.com.

Unit testing in nf-test

Within the context of a Nextflow pipeline, unit testing involves testing a single process, workflow, or function in isolation. We developed a domain-specific language (DSL) based on Groovy that offers methods and keywords to describe the expected behavior of any Nextflow unit. A project typically consists of different test suites, with one test suite per test subject (e.g., process, workflow, or pipeline). Each test suite contains one or more test cases to describe the expected behavior of the test subject. A test case is defined with the "test" keyword followed by two distinct blocks: (1) the "when" block, which sets the input parameters of the test subject, and (2) the "then" block, which describes the expected output channels of the test subject when executed with the input parameters defined in the "when" block. Typically, the "then" block mainly contains assertions to check assumptions, such as the content of an output channel or files. Several functions are provided for writing assertions and simplifying Nextflow channel testing. Additionally, the "then" block accepts any Groovy script and allows the import of third-party Java libraries.

This modularity enables writing integration tests for a sub-workflow to ensure that the processes work together as expected. Thus, testing is consistently conducted using the same

syntax and concepts throughout the entire Nextflow project. Together, these different levels of testing provide a comprehensive and effective test strategy for the pipeline's functionality, ensuring that all components work together as intended (see **Figure 2**).

Test case execution

One or more DSL files serve as inputs for nf-test, which automatically executes the entire suite of tests. For each test, the runner automatically creates a Nextflow driver script that (a) initiates the Nextflow unit with parameters defined in the "when" block, (b) executes the unit, and (c) serializes all output channels. Subsequently, it parses the content of the output channels and evaluates the assertions defined in the "then" block to verify if the output aligns with the expected behavior. As processes are executed in parallel, Nextflow channels emit output values in a random order. Deterministic assertions are enabled by sorting the Nextflow channel tuples. The sorting is performed automatically by nf-test prior to the launch of the "then" closure. Finally, the test results are aggregated and reported in various formats (e.g., JUnit, XML, TAP [http://testanything.org/] or CSV), allowing for processing by third-party reporting tools (see **Figure 3a**).

Smart testing and parallelization

nf-test provides a strategic approach called *smart testing* to optimize test execution time by minimizing the number of tests executed. We implemented a graph-based approach inspired by firewall testing [14, 15], to select and prioritize tests based on their relevance to recent modifications. This enables nf-test to identify and execute tests for the impacted files and avoids redundantly retesting of unaffected components. As most Nextflow pipelines take advantage of a version control system, we have integrated Git (https://git-scm.com) support to automatically detect changes in the local working tree. This makes it possible to test changes between commits or branches, ensuring continuous validation of pipeline modifications on each commit or between releases.

Parallelization is achieved through the implementation of *test list sharding* [16]. This technique divides the test suite into smaller subsets and distributes them across multiple parallel execution environments or cores. To optimize test distribution, we provide two distinct strategies: (a) a simple chunking algorithm that splits the test list into chunks, and (b) a round-robin approach for equitable allocation.

Snapshot testing

We adapted the main idea of snapshot testing and applied it to the output of pipelines. Snapshot testing is a technique commonly utilized in web development to prevent unexpected changes in a user interface [17]. nf-test captures a snapshot of output channels or any other provided objects. These snapshots are then compared against reference snapshot files that are stored alongside the respective tests. If the two snapshots do not match, the test will fail (see **Figure 3b**). If the change is unexpected, the user can address the bug detected by the test. Otherwise, the reference snapshot needs to be updated to the new output by rerunning nf-test with a special option (`--update-snapshot`). All snapshot files are created automatically in the background and are optimized for large files and complex objects.

Extensions for bioinformatics

nf-test provides a plugin system for reusing code snippets, saving development time, making test code cleaner and enhancing maintainability. The plugin system, based on Groovy, is well-documented to encourage users to create and share their own extensions. For example, in most bioinformatics files formats, file determinism is not always guaranteed, since timestamps or input filenames may prevent files from being byte-identically. In these cases, MD5 sums cannot be used and validating the dynamic output content can be time-intensive. At the time of writing this paper, we provide plugins for validating VCF, BAM, FASTA, FASTQ, and CSV files (see https://plugins.nf-test.com).

Resource saving through smart testing

First, we evaluated smart testing and its impact on the execution time using the nf-core/fetchngs pipeline. This pipeline implemented 50 test cases for 17 components. The dependency graph illustrates the connections and dependencies between modules, workflows and test cases. Given that each component includes at least one test case, the pipeline achieves 100% coverage (see **Figure S1**). The total execution time for running all tests is 1,122 seconds. As hypothesized, we observed that pipeline end-to-end tests show the slowest performance (see **Table 1**). We simulated different modifications to assess the impact of changes, varying the number of modified files and different types of changes: (a) changes to the logic of the module itself and (b) changes to the module interface (e.g. adding a new input channel). The results show that smart testing saves between 46% and 80% of the execution time by minimizing the number of executed test cases (see **Table 2**). We reviewed the test results from a full run of the entire test suite and confirmed that all changes are detected by our approach.

Second, we analyzed the last 500 commits within the nf-core/modules project, spanning from October 26, 2023 to February 23, 2024. At the time of writing this paper, nf-core/modules contains 1,150 modules, and 56 workflows as well as more than 800 test cases were implemented by the community. As expected, most of the commits and pull requests (PRs) affect only one module. In such cases, the time savings are significantly higher, as it only requires testing the relevant unit tests and potential integration tests of workflows. nf-test was able to accurately identify the specific test cases and integration tests required for the committed changes (see **Figure 4a**). Approximately 30% of the commits required the execution of more than 25 test cases, with most of these commits being refactoring or restructuring tasks (see **Figure 4b**). The total number of executed test cases could be reduced from 238,205 to 1,600. nf-test parsed and analyzed 1,560 unique files in under 1 second to construct the dependency graph.

Execution time reduction through parallelization

We evaluated the efficiency and speedup of parallelization using nf-gwas [18], a pipeline for running genome-wide association studies, which consists of multiple long-running end-to-end tests (see **Figure S2** for the dependency graph). Initially, executing the 49 test cases on a single machine required 1,718 seconds. The execution time was reduced to 487 seconds by distributing the workload across five machines, achieving a speedup of 3.5. The default strategy distributes tests by name, which may result in unbalanced execution times, especially when outliers are present (see **Table 3**). Using a round-robin approach further reduced the time to 333 seconds, resulting in a speedup of 5.2.

Code reduction through snapshot testing

We analyzed how snapshot testing in nf-test can improve quality and maintainability by minimizing the effort required for writing manual assertions. Writing manual assertions for each output item can be time-intensive and error-prone, especially in pipelines with extensive outputs. For example, the nf-gwas pipeline generates five output files for a single phenotype. Writing a simple regression test involves creating an assertion for each file to verify its existence and another to ensure its content matches expectations. This manual process implies generating an MD5 hash for each file and using it in the corresponding assert block, resulting in 15 lines of code for three phenotypes. Additionally, this test case must be updated whenever the pipeline generates additional output files, requiring manual synchronization. Snapshot testing streamlines this process by replacing 15 lines of code with a single line. nf-test automatically creates the MD5 sum for each of these files on the first run and provides commands to update the reference snapshot.

We adapted the nf-gwas pipeline and simulated various software updates for REGENIE [19], the underlying software: (a) changes in the output file format (renaming the column "LOG10P" to "PVALUE"), (b) a bug in association detection (where six variants are no longer genome-

wide significant), and (c) modifications to default parameters. Change (a) will break the pipeline and is easy to detect without test cases by running the pipeline with test data. However, changes (b) and (c) lead to incorrect output results without breaking the pipeline. Thus, these are the worst-case scenarios for a pipeline developer. The implemented test cases were able to detect all of these issues. For example, the unit test for REGENIE that checks if 116 variants are genome-wide significant fails, as only 110 variants were found. Similarly, changes in default parameters (e.g., a more restricted MAF filter) lead to a different number of resulting variants and causing the test to fail.

DISCUSSION

Since its first release in October 2021, nf-test has been integrated into dozens of pipelines and has been downloaded over 120,000 times. As the new standard testing framework in nf-core for both pipelines and provided modules, it shows a high level of acceptance, reflecting the community's awareness of the importance of testing pipelines.

There have been various efforts to test Nextflow pipelines in the past, including NFTest [13] and the nf-core framework [3], which utilized pytest-workflow (https://pytest-workflow.readthedocs.io). However, these solutions are based on YAML files with predefined assertions, are limited to end-to-end testing, relied on external scripts to validate output files and do not support optimization strategies like parallelization and smart testing (see **Table 4**). nf-test addresses these gaps by offering a dedicated testing framework that simplifies the process of writing, executing, and analyzing tests for Nextflow pipelines. It offers a DSL that follows a similar naming and philosophy as Nextflow DSL 2 and enables writing complex assertions that are often needed to validate the huge output of bioinformatics analysis. As the DSL is based on Groovy, it can be extended by users themselves, and they can rely on a rich ecosystem of Java/JVM libraries in the field of bioinformatics. In addition, sharing domain specific assertions through plugins facilitates collaboration among users.

We implemented a unit testing approach where all components of a Nextflow pipeline can be tested without the need for manually writing additional Nextflow workflows to execute a subprocess with test data. This modularity also facilitates writing integration tests for a sub-workflow to ensure that the processes work together as expected. All testing is conducted consistently across a project, and any possible introduced side effects are detected. This modularity not only simplifies the debugging process but also encourages iterative development and code reuse. Each module can be individually tested and seamlessly integrated into the final pipeline or workflow composition. Especially, when using large module libraries like the ones provided by nf-core/modules, integration tests are important to ensure that any third-party update doesn't break its own pipeline.

In pipelines with extensive outputs, writing manual assertions for each output item can be time-intensive and error-prone. To mitigate this challenge, we introduced snapshot testing as a complementary approach. Instead of specifying individual assertions for each output, snapshots capture the state of the output channels or folders, including file names and hash values. These snapshots are automatically generated during the first run, and nf-test is able to compare the current snapshot with the expected reference snapshot during every subsequent run. This makes regression testing more efficient and helps to catch regressions early in the development cycle. Moreover, it ensures that results remain reproducible and consistent across different runs of the same pipeline and after software updates. By evaluating the nf-gwas pipeline, we demonstrated how nf-test and snapshot testing could enhance code quality and maintainability by minimizing the effort of writing manual assertions.

Regression testing involves retesting the pipeline after any code modification. Given that bioinformatics pipelines process large input data and utilize complex algorithms, the execution of full regression tests could take hours. To save effort and time, nf-test only needs to retest those tests affected by the modification. We demonstrated, using the example of the nf-core/modules project, that the majority of changes and commits in such large projects affect

only a specific set of modules and tests. Therefore, nf-test implements various approaches to detect minimal sets of tests that need to be rerun. This results in resource savings and faster development cycles (up to 80%).

When the number of tests in a pipeline is large and the execution time becomes long, test-list sharding can be used to split the tests across several machines. The experimental results indicated that this approach enables a significant performance gain of up to 80% in terms of reduced execution time on five machines. However, there is no guarantee of an optimal or fair split among resources as the splitting decision is not influenced by data from previous runs. The implemented round-robin strategy simply attempts to distribute the workload evenly. Nonetheless, the setup remains easy since no shared database, queuing system or orchestration instance is needed. Combined with the integration of Git, nf-test enables the setup of Test-Driven Development (TDD) and Continuous Integration (CI) for Nextflow pipelines.

The implemented dependency analysis provides an overview of pipeline coverage and quantifies the testing effort. However, the current implementation has limitations, as it only reflects if at least one test case per unit exists, without indicating whether all instructions or branches are covered by any test case. In future work, we aim to extend this approach and to include metrics that reflect the complexity of a change set. Moreover, nf-test depends on local environments, which can make it less adaptable to certain infrastructures. For example, running test cases on different cloud providers is currently limited and will be addressed in future versions.

DATA AVAILABILITY

nf-test and its documentation are available at https://www.nf-test.com. The source code is available at https://github.com/askimed/nf-test and is released under the MIT license.

ACKNOWLEDGMENTS

We would like to express our gratitude to the nf-core community for their support in this project, including their contributions of test cases and valuable suggestions. Special thanks go to Sateesh Peri, Maxime Garcia, Edmund Miller, Nicolas Vannieuwkerke, Harshil Patel, Adam Talbot, and all GitHub contributors.

AUTHOR CONTRIBUTIONS

LF conceptualized and supervised the project. LF and SS implemented the software. LF and SS conducted formal analyses and wrote the manuscript (original draft, review and editing).

CONFLICT OF INTEREST

None declared.

FIGURES

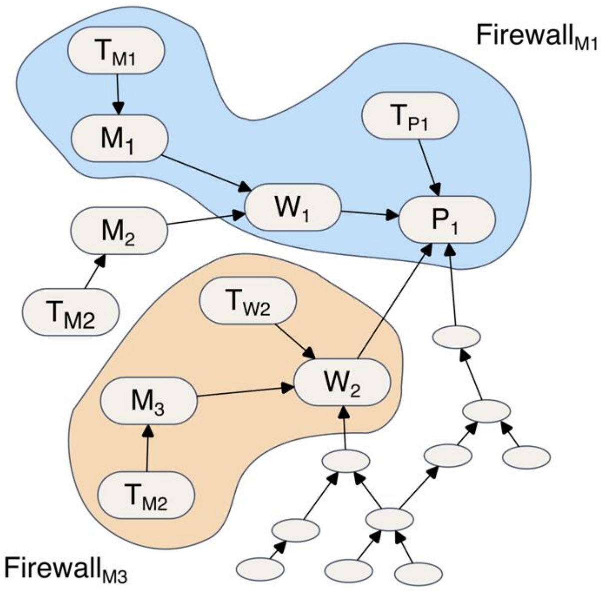

Figure 1: Example of a dependency graph and firewalls. The figure illustrates the dependencies of tests (*T_x*), modules/processes (*M_x*), workflows (*W_x*), and pipelines (*P_x*) within a Nextflow project. Changes to module *M_1* will only affect test cases inside Firewall *M_1*. Firewall *M_3* is more compact because workflow *W_2* contains a test case ensuring the integrity of *M_3*. This approach avoids running expensive end-to-end tests for *P_1*.

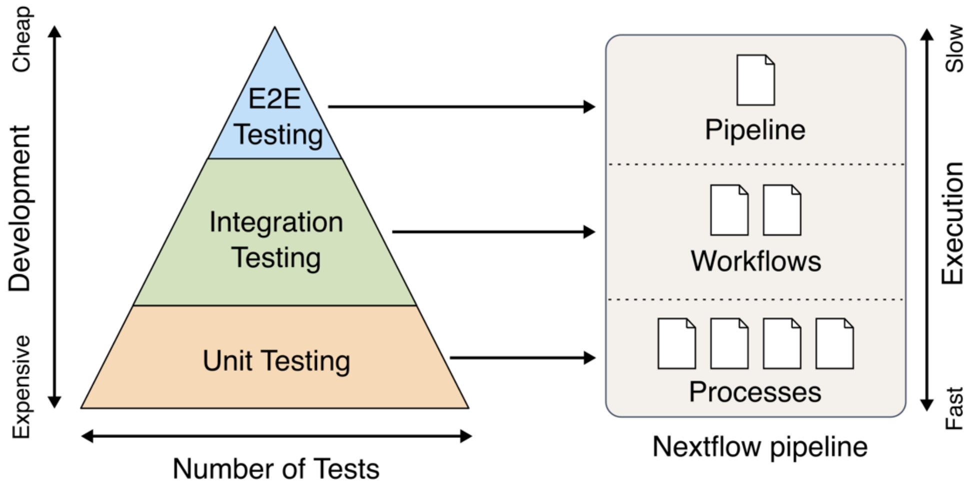

Figure 2: Overview of different test strategies provided by nf-tet. A comprehensive and efficient test strategy for a Nextflow project includes unit, integration, and end-to-end testing.

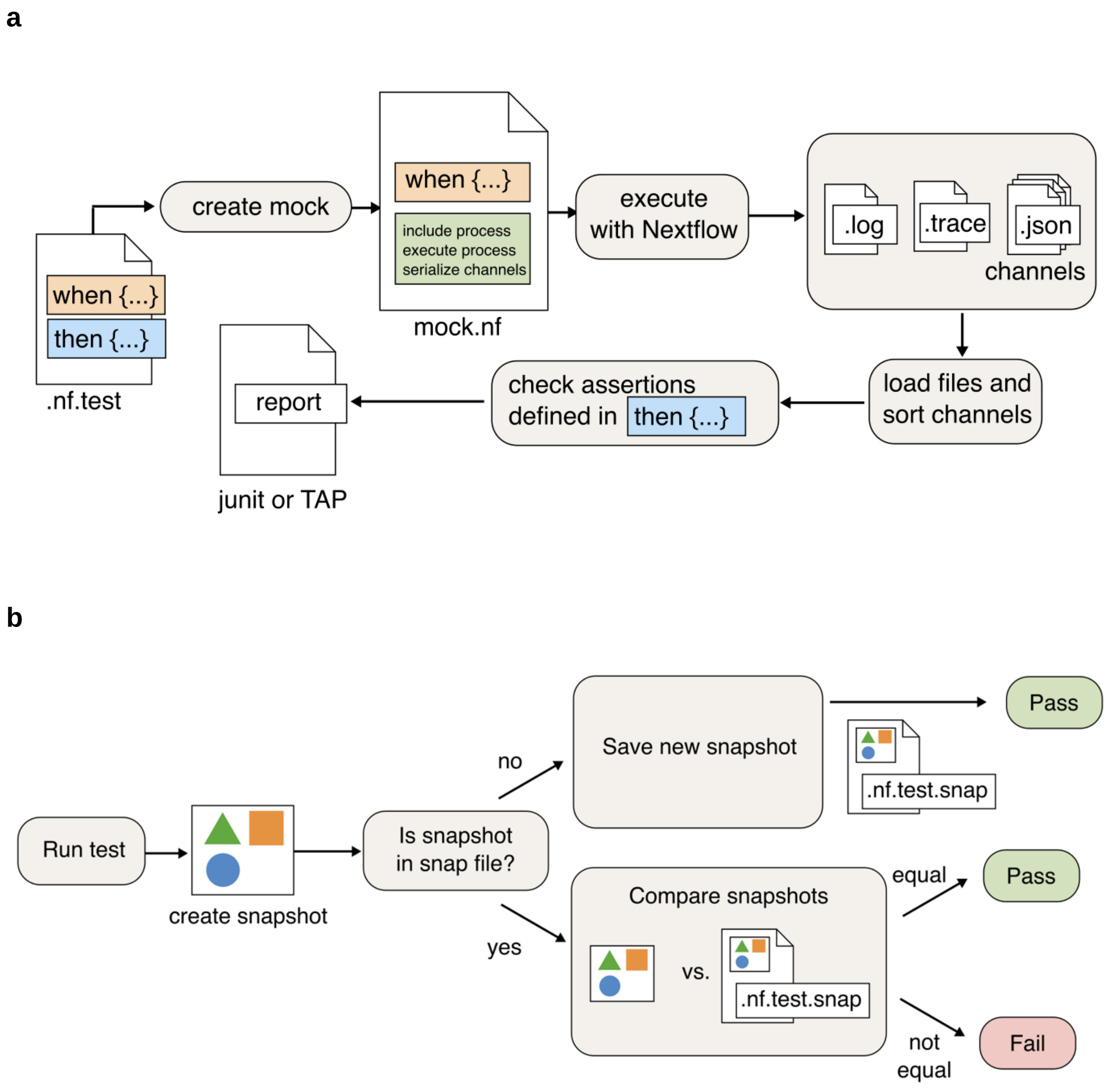

Figure 3: Architecture of the implemented test framework. (a) nf-test generates Nextflow scripts for tests: (i) initializes Nextflow unit with “when” block parameters, (ii) executes the unit and serializes output channels, (iii) parses channel content, evaluates “then” block assertions for output validation. Test results are aggregated and reported in multiple formats. **(b)** nf-test runs a test, creates and compares output objects against reference snapshot files stored with the tests. A test fails if the snapshots don't match, indicating either unexpected changes or the need to update the reference snapshot to reflect new outputs.

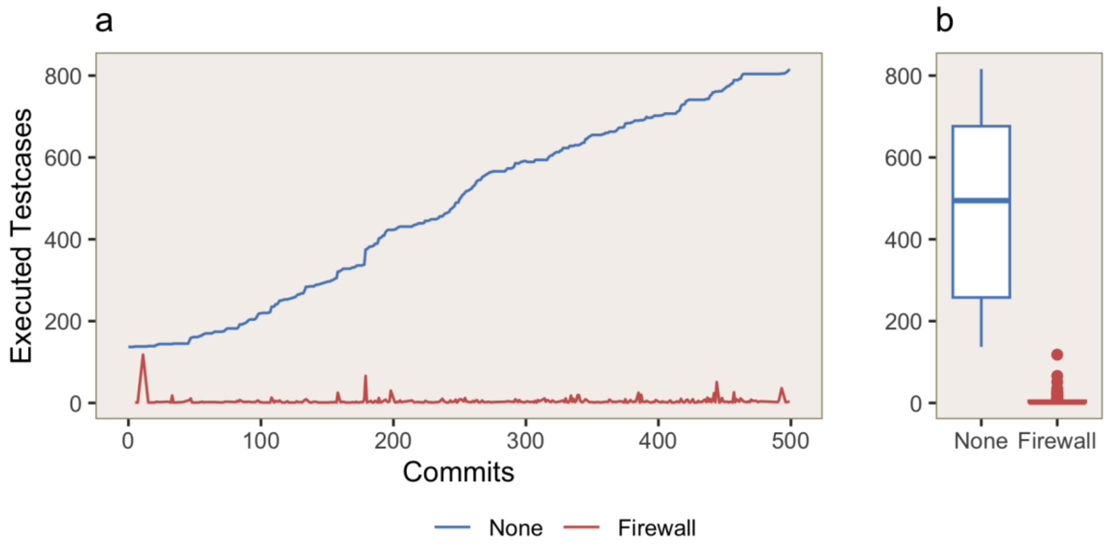

Figure 4: Last 500 commits of the nf-core/modules projects between 2023/10/26 and 2024/02/23. (A) The blue line represents the number of test cases that would be executed without any optimization strategy. The red line depicts the number of executed test cases when using the implemented Firewall strategy. (B) Boxplot of the number of executed test cases per commit.

TABLES

Table 1: Number of test cases in the nf-core/fetchngs pipeline (Version 1.12.0). Each of the 17 components has at least one test case, for a total of 50 test cases. The total execution time is 1,122 sec.

			Execution Time	
	Tests suites	Tests cases	Mean (sec)	Total (sec)
Functions	3	14	2.6	36.5
Pipelines	1	1	512.1	512.1
Modules/Processes	10	13	11.2	145.7
Workflows	15	22	19.4	427.8
Total	29	50	-	1,122.0

Table 2: Time and resource saving for different modifications of the nf-core/fetchngs. We simulated several typical modifications and measured the execution time using nf-test's optimization strategy. Time savings areis calculated based on the execution time of a full run (1,122 sec).

		Execution Time		
Modification	Executed test cases	Mean (sec)	Total (sec)	Saving
changed module sra_to_samplesheet	10	22.3	222.5	80.2%
changed modules sra_to_samplesheet multiqc_mappings_config	11	52.7	579.4	48.4%
update of a nf-core module: utils_nfcore_pipeline	23	14.4	332.1	70.4%
changed main workflow main.nf`	1	596.3	596.3	46.8%

Table 3: Time and speedup for different sharding strategies of the nf-gwas pipeline.

Strategy	Shards	Median (sec)	Total Time	Speed Up
none	5	368 +/-164	487 sec	3.52
Round-Robin	5	314 +/- 10	333 sec	5.15

Table 4: Comparison of nf-test with similar approaches.

	nf-test	NFTest	pytest-workflow
**End to End Testing**	Yes	Yes	Yes
**Unit Testing**	Yes	Manual writing Nextflow script	Manual writing Nextflow script
**Snapshot Testing**	Yes	Manual preparation of expected output files	Manual preparation of expected output files
**Optimized test strategies**	Yes	No	No
**Dependency analysis**	Yes	No	No
**Coverage reporting**	Yes	No	No
**Tags**	Yes	No	Yes
**Output formats**	Junit, xml, TAP and csv	No	Junit, html
**Code generation**	config file and for each unit	config file	Using nf-core tools
**Custom assertions**	Yes	Through external third party scripts	No
**Bioinformatics support**	Yes (e.g. vcf, fasta, …)	No	No
**Parallelization**	Sharding	No	No
**Modularity and portability**	Each unit has its own test files that can be transferred	One test file for whole project	Multiple files
**Writing test cases**	Extendable DSL	YAML with predefined structure	YAML with predefined structure
**License**	MIT	GPL-2.0	AGPL-3.0
**Website**	https://www.nf-test.com	https://github.com/uclahs-cds/tool-NFTest	https://pytest-workflow.readthedocs.io

REFERENCES

1. Kumar P, Paul RK, Roy HS, Yeasin M, Ajit, Paul AK: **Big Data Analysis in Computational Biology and Bioinformatics**. *Methods Mol Biol* 2024, **2719**:181-197.
2. Di Tommaso P, Chatzou M, Floden EW, Barja PP, Palumbo E, Notredame C: **Nextflow enables reproducible computational workflows**. *Nat Biotechnol* 2017, **35**(4):316-319.
3. Ewels PA, Peltzer A, Fillinger S, Patel H, Alneberg J, Wilm A, Garcia MU, Di Tommaso P, Nahnsen S: **The nf-core framework for community-curated bioinformatics pipelines**. *Nat Biotechnol* 2020, **38**(3):276-278.
4. Langer BE, Amaral A, Baudement M-O, Bonath F, Charles M, Chitneedi PK, Clark EL, Di Tommaso P, Djebali S, Ewels PA *et al*: **Empowering bioinformatics communities with Nextflow and nf-core**. *bioRxiv* 2024:2024.2005.2010.592912.
5. Djaffardjy M, Marchment G, Sebe C, Blanchet R, Bellajhame K, Gaignard A, Lemoine F, Cohen-Boulakia S: **Developing and reusing bioinformatics data analysis pipelines using scientific workflow systems**. *Comput Struct Biotechnol J* 2023, **21**:2075-2085.
6. van der Putten BCL, Mendes CI, Talbot BM, de Korne-Elenbaas J, Mamede R, Vila-Cerqueira P, Coelho LP, Gulvik CA, Katz LS, The Asm Ngs Hackathon P: **Software testing in microbial bioinformatics: a call to action**. *Microb Genom* 2022, **8**(3).
7. Electrical Io, Engineers E: **Software Engineering Standards: ANSI/IEEE Std 729-1983, Glossary of Software Engineering Terminology**: Inst. of Electrical and Electronics Engineers; 1984.
8. Piras ME, Pireddu L, Zanetti G: **wft4galaxy: a workflow testing tool for galaxy**. *Bioinformatics* 2017, **33**(23):3805-3807.
9. Krafczyk M, Shi A, Bhaskar A, Marinov D, Stodden V: **Scientific Tests and Continuous Integration Strategies to Enhance Reproducibility in the Scientific Software Context**. In: *Proceedings of the 2nd International Workshop on Practical Reproducible Evaluation of Computer Systems; Phoenix, AZ, USA*. Association for Computing Machinery 2019: 23–28.
10. Beizer B: **Software testing techniques (2nd ed.)**: Van Nostrand Reinhold Co.; 1990.
11. Kamali AH, Giannoulatou E, Chen TY, Charleston MA, McEwan AL, Ho JWK: **How to test bioinformatics software?** *Biophys Rev* 2015, **7**(3):343-352.
12. Ferenc K, Rauluseviciute I, Hovan L, Kumar V, Kuijjer ML, Mathelier A: **Improving bioinformatics software quality through teamwork**. *Bioinformatics* 2024, **40**(11).
13. Patel Y, Zhu C, Yamaguchi TN, Bugh YZ, Tian M, Holmes A, Fitz-Gibbon ST, Boutros PC: **NFTest: automated testing of Nextflow pipelines**. *Bioinformatics* 2024, **40**(2).
14. White LJ, Leung HK: **A firewall concept for both control-flow and data-flow in regression integration testing**. In: *Proceedings Conference on Software Maintenance 1992: 1992*. IEEE Computer Society: 262,263,264,265,266,267,268,269,270,271-262,263,264,265,266,267,268,269,270,271.
15. Leung HK, White L: **A study of integration testing and software regression at the integration level**. In: *Proceedings Conference on Software Maintenance 1990: 1990*. IEEE: 290-301.

16. Gonsalves M, Mandala J: **Regression Test List Sharding in a Distributed Test Environment**. In: *E3S Web of Conferences: 2023*. EDP Sciences: 04028.

17. Gazzinelli Cruz VP, Rocha H, Valente MT: **Snapshot testing in practice:: Benefits and drawbacks**. 2023.

18. Schonherr S, Schachtl-Riess JF, Di Maio S, Filosi M, Mark M, Lamina C, Fuchsberger C, Kronenberg F, Forer L: **Performing highly parallelized and reproducible GWAS analysis on biobank-scale data**. *NAR Genom Bioinform* 2024, **6**(1):lqae015.

19. Mbatchou J, Barnard L, Backman J, Marcketta A, Kosmicki JA, Ziyatdinov A, Benner C, O'Dushlaine C, Barber M, Boutkov B *et al*: **Computationally efficient whole-genome regression for quantitative and binary traits**. *Nat Genet* 2021, **53**(7):1097-1103.

Supplementary Material

Click here to access/download
Supplementary Material
supplemental-material.pdf
