# Supplementary material for: Improving the reliability, quality, and maintainability of bioinformatics pipelines with nf-test
Source: Gigascience. 2025 Oct 22;14:giaf130. doi: 10.1093/gigascience/giaf130 (PMC12616847; doi:10.1093/gigascience/giaf130)
Supplement: giaf130_GIGA-D-25-00116_Revision_1 [file giaf130_giga-d-25-00116_revision_1.pdf]

GigaScience

Improving the Reliability, Quality and Maintainability of Bioinformatics Pipelines with nf-test

--Manuscript Draft--

**Manuscript Number:**	GIGA-D-25-00116R1
**Full Title:**	Improving the Reliability, Quality and Maintainability of Bioinformatics Pipelines with nf-test
**Article Type:**	Research
**Funding Information:**	
**Abstract:**	The workflow management system Nextflow, together with the nf-core community, has established an essential ecosystem in bioinformatics. However, ensuring the correctness and reliability of large and complex Nextflow pipelines remains challenging due to the lack of a unified, automated unit-testing framework. To address this gap, we present nf-test, a modular testing framework for bioinformatics workflows. It enables users to test process blocks, workflow patterns, and entire pipelines in isolation while validating their outputs. Built with a syntax similar to Nextflow DSL2, nf-test offers unique features such as snapshot testing and smart testing, which optimize resource usage by testing only modified modules. We demonstrate across multiple pipelines that these features minimize development time, reduce test execution time by up to 80%, and enhance software quality by identifying bugs and issues early in the development process. Already adopted by numerous pipelines, nf-test significantly improves the robustness, maintainability and reliability of bioinformatics pipelines.
**Corresponding Author:**	Lukas Forer<br>Medical University of Innsbruck: Medizinische Universitat Innsbruck<br>Innsbruck, AUSTRIA
**Corresponding Author Secondary Information:**	
**Corresponding Author's Institution:**	Medical University of Innsbruck: Medizinische Universitat Innsbruck
**Corresponding Author's Secondary Institution:**	
**First Author:**	Lukas Forer
**First Author Secondary Information:**	
**Order of Authors:**	Lukas Forer
	Sebastian Schönherr
**Order of Authors Secondary Information:**	
**Response to Reviewers:**	The reviewer point-by-point response has been uploaded as a separate PDF file to improve readability (see Supplementary Material).
**Additional Information:**	
**Question**	**Response**
Are you submitting this manuscript to a special series or article collection?	No
**Experimental design and statistics**	Yes

Powered by Editorial Manager® and ProduXion Manager® from Aries Systems Corporation

Full details of the experimental design and statistical methods used should be given in the Methods section, as detailed in our Minimum Standards Reporting Checklist. Information essential to interpreting the data presented should be made available in the figure legends.<br><br>Have you included all the information requested in your manuscript?	
**Resources**<br><br>A description of all resources used, including antibodies, cell lines, animals and software tools, with enough information to allow them to be uniquely identified, should be included in the Methods section. Authors are strongly encouraged to cite Research Resource Identifiers (RRIDs) for antibodies, model organisms and tools, where possible.<br><br>Have you included the information requested as detailed in our Minimum Standards Reporting Checklist?	Yes
**Availability of data and materials**<br><br>All datasets and code on which the conclusions of the paper rely must be either included in your submission or deposited in publicly available repositories (where available and ethically appropriate), referencing such data using a unique identifier in the references and in the "Availability of Data and Materials" section of your manuscript.<br><br>Have you have met the above requirement as detailed in our Minimum Standards Reporting Checklist?	Yes
GigaScience has policies and guidelines	No

in place for the use of generative AI-writing tools such as ChatGPT. If you have used such writing tools to assist with writing the manuscript this must be declared and cited in the text. Authors should not list AI-writing tools and other AI-assisted technologies as an author or co-author and should acknowledge that they are fully responsible for text generated or refined by AI-writing tools.<p><br><br>A summary of use (particularly in the introduction or among methods) needs to be included at the end of the paper, and the outputs should also be included as a supplementary file hosted in GigaDB or other open repositories. Please read our guidelines for more information. <p><br><br>By submitting to GigaScience, you are aware of the journal's AI-writing tools policy, and if you have declared use of such tools below, you have acknowledged this where appropriate in your manuscript and have made a summary of use and outputs available. <p><br><b>AI-assisted writing tools have been used in the preparation of this manuscript?	

main text Click here to access/download;Manuscript;manuscript_Oct 20.docx

Improving the Reliability, Quality and Maintainability of Bioinformatics Pipelines with nf-test

Lukas Forer[1*] and Sebastian Schönherr[1]

1. Institute of Genetic Epidemiology, Medical University of Innsbruck, Innsbruck, Austria

* Corresponding Author:

Lukas Forer, PhD.

lukas.forer@i-med.ac.at

Institute of Genetic Epidemiology

Medical University of Innsbruck

Schöpfstrasse 3

6020 Innsbruck, Austria

ORCID iDs: Lukas Forer [0000-0003-2139-7329]; Sebastian Schönherr [0000-0001-5909-9226]

ABSTRACT

The workflow management system Nextflow, together with the nf-core community, has established an essential ecosystem in bioinformatics. However, ensuring the correctness and reliability of large and complex Nextflow pipelines remains challenging due to the lack of a unified, automated unit-testing framework. To address this gap, we present nf-test, a modular testing framework for bioinformatics workflows. It enables users to test process blocks, workflow patterns, and entire pipelines in isolation while validating their outputs. Built with a syntax similar to Nextflow DSL2, nf-test offers unique features such as snapshot testing and smart testing, which optimize resource usage by testing only modified modules. We demonstrate across multiple pipelines that these features minimize development time, reduce test execution time by up to 80%, and enhance software quality by identifying bugs and issues early in the development process. Already adopted by numerous pipelines, nf-test significantly improves the robustness, maintainability and reliability of bioinformatics pipelines.

Keywords: Nextflow, Pipeline Testing, Test Automation

INTRODUCTION

The large volumes of biological data generated across genomics, proteomics, or metabolomics, have transformed bioinformatics and computational biology into big data sciences [1]. These disciplines require not only the processing of massive datasets but also the application of complex workflows to analyze, filter, and transform data to uncover complex biological relationships. Nextflow [2] has emerged as a powerful and flexible platform for building scalable and reproducible computational pipelines. Together with nf-core [3], a community-driven initiative dedicated to developing and maintaining best-practice pipelines, Nextflow has become a rich ecosystem for pipeline development [4]. However, as pipeline complexity grows, as demonstrated by several nf-core pipelines, ensuring their correctness and reliability becomes a critical challenge, especially when incorporating new features without disrupting existing functionality.

Automated testing is the process of evaluating and verifying that a software product performs as intended [5]. It is essential in scientific pipeline development to confirm functionality and ensure accurate data processing and analysis [6, 7]. The process involves defining test objectives, selecting test datasets, creating test cases, executing the pipeline with these cases and verifying the test results [8]. Many bioinformatics pipelines face the so-called oracle problem, as they often process large input and output datasets while implementing complex algorithms without a clear gold standard [9]. This complexity makes writing test cases difficult and time-consuming. Consequently, most bioinformatics software lacks established software development quality standards [10]. An effective and comprehensive testing strategy for a pipeline's functionality should cover multiple levels of testing, including unit testing, integration testing, and end-to-end testing. Although testing is a critical aspect of scientific software development [11], it remains underused [12]. Consequently, Nextflow pipeline maintenance demands substantial time and effort to ensure that outputs remain consistent with prior versions and scientifically valid when benchmarked against a ground truth.

Despite efforts by existing solutions to automate end-to-end testing of Nextflow pipelines [13], as well as a Python-based workflow developed by nf-core [3], a unified and robust unit-style testing framework tailored to large and complex Nextflow pipelines is still lacking. This limits the efficient and automated validation of their functionality, making it difficult for users to ensure workflow accuracy and potentially leading to errors or artifacts in data analysis and interpretation. This issue becomes even more critical when considering the clinical utility of such pipelines. Furthermore, long execution times prevent developers from rerunning tests promptly, thereby reducing productivity.

Here, we present nf-test, a testing framework designed to address these challenges within the context of Nextflow pipelines. nf-test provides a domain-specific language with a syntax similar to Nextflow DSL2 to describe the expected behavior and output data of a process or workflow. It introduces a modular approach that enables developers to isolate and validate individual process blocks, workflow patterns, and even entire pipelines. This modularity not only simplifies debugging but also promotes iterative development and code reuse. Additionally, nf-test incorporates snapshot testing and several optimization strategies to make testing data-intensive pipelines more efficient. These features help pipeline developers catch issues early in the development cycle, enabling a robust and agile development process that results in more reliable pipelines. Serving as the new standard testing framework for nf-core [3], nf-test has become an essential tool for pipeline developers. It is freely available, with extensive documentation provided on the website [14].

MATERIAL AND METHODS

Design and Implementation

nf-test is implemented in Java as a command-line program, shares the same requirements as Nextflow, and is compatible with Linux and macOS. We adapted well-established testing concepts from software and web development for use in Nextflow pipeline testing. nf-test is built on a modular architecture and utilizes a plugin system, enabling effortless extension with new output formats, assertions, and optimization strategies. The software offers a wide range of project-specific configuration options and can be easily installed on continuous integration (CI) platforms using the provided installation script. Instructions, user guides, and examples are available at [14]. All code is open-source and freely available under the MIT License.

Unit, Integration, and End-to-End Testing

Within the context of a Nextflow pipeline, unit testing involves evaluating a single process, workflow, or function in isolation. We developed a domain-specific language (DSL) based on Groovy that provides methods and keywords to describe the expected behavior of any Nextflow unit. A project typically comprises multiple test suites, with one test suite per test subject (e.g., process, workflow, or pipeline). Each test suite contains one or more test cases that specify the expected behavior of the test subject. A test case is defined using the "test" keyword, followed by two distinct blocks: (1) the "when" block, which sets the input parameters of the test subject, and (2) the "then" block, which defines the expected output channels when the test subject is executed with the input parameters from the "when" block. Typically, the "then" block primarily contains assertions to verify assumptions, such as the content of an output channel or files. Several built-in functions simplify writing assertions and testing Nextflow channels. Additionally, the "then" block supports any Groovy script and allows the import of third-party Java libraries.

This modularity also enables writing integration tests for sub-workflows, ensuring that individual processes interact as expected. Similarly, end-to-end tests are written in the same way, with the “when” block defining the user-provided input parameters and the “then” block verifying the expected output files. Consequently, testing is conducted consistently using the same syntax and concepts throughout the entire Nextflow project. Together, these different levels of testing provide a comprehensive and effective strategy for validating pipeline functionality, ensuring that all components work together as intended (see **Figure 1**).

Test case execution

One or more DSL files serve as inputs for nf-test, which automatically generates the entire suite of tests. For each test, the runner automatically creates a Nextflow driver script that (a) initializes the Nextflow unit with parameters defined in the “when” block, (b) executes the unit, and (c) serializes all output channels. The runner then parses the output channels and evaluates the assertions defined in the "then" block to verify whether the output matches the expected behavior. Since processes are executed in parallel, Nextflow channels emit output values in a random order. nf-test ensures deterministic assertions by automatically sorting channel tuples. Finally, test results are aggregated and reported in multiple formats (e.g., JUnit, XML, TAP [15] or CSV), enabling processing by third-party reporting tools (see **Figure 2a**).

Dependency Graph and Smart Testing

nf-test constructs a dependency graph that captures the dependencies among all modules, workflows, and test suites within a given project. In this graph, nodes represent individual modules, workflows and test suites, while edges represent their dependencies. To build the graph, the algorithm traverses the entire project directory, identifies connections between different components (such as by parsing “include” statements), and maps them onto the graph structure. **Figure 3** illustrates this concept: nodes represent processes (M_1, M_2 and M_3),

workflows (W_1 and W_2) and pipelines (P_1), while edges denote their dependencies (for example, W_1 depends on processes M_1 and M_2). Test suites are linked to the components they validate (e.g., T_{M1} is the test suite for M_1). Since a test suite may also depend on data or configuration files, nf-test allows users to (a) define files that always trigger a full retest (e.g., Dockerfile), and (b) specify test assets that are automatically added to the dependency graph (e.g., input data). This directed graph provides insights into the pipeline's architecture, supporting a comprehensive understanding of its dependencies and interactions. Building on the dependency graph, nf-test introduces a strategy called smart testing to optimize test execution time by minimizing the number of tests run. The core idea is to use the dependency information to identify a minimal set of tests sufficient to detect regressions in modified processes or workflows. This improves efficiency, since only relevant test suites are executed. Inspired by a concept proposed by Leung and White [16, 17], nf-test implements a *firewall strategy*. When a module changes, nf-test identifies all affected nodes and constructs a firewall around them, containing only the necessary tests. Only these tests need to be retested. For example, in **Figure 3**, $Firewall_{M1}$ includes all nodes marked for retesting when module *M1* is modified. Tests associated with M_1 are added first, followed by the integration tests for W_1, which depends on M_1. Since W_1 has no direct tests, we trace its dependents, in this case, P_1. Because P_1, has a test case, it is added to the firewall and reused to indirectly validate *W1*. By contrast, if M_3 is modified, the firewall is smaller, as W_2 has its own test case and T_{P1} does not need to be included. This approach enables fine-grained control over the firewall, allowing expensive end-to-end tests to be excluded where appropriate, while maintaining accuracy and reducing testing costs.

Git Support and Testing Status

As most Nextflow pipelines use version control, nf-test also integrates with Git [18] to automatically detect changes in the working tree. This enables test execution on differences

between commits or branches, ensuring continuous validation of pipeline modifications across commits and releases.

The testing status is determined by analyzing the dependency graph to identify which components are directly or indirectly verified by at least one test case. This provides an overview of which parts of the workflow are exercised by tests, without implying complete or line-level testing. Additionally, the testing status can be computed for a firewall to provide a safety-related status metric.

Parallelization with Test List Sharding

Parallelization is achieved through test list sharding [19], which splits the test suite into smaller subsets and distributes them across multiple parallel execution environments or machines. Discovered tests are sorted by type and filename to ensure a deterministic order across machines. To optimize test distribution across n machines, nf-test provides two strategies: (a) a simple chunking algorithm that splits the test list into n chunks and (b) a round-robin approach for equitable allocation *("--shard-strategy round-robin"*). For example, splitting a suite into three shards can be done by running one of the following commands on each machine: "*nf-test --shard 1/3", "nf-test --shard 2/3", and "nf-test --shard 3/3"*.

Snapshot Testing

Snapshot testing is a technique commonly utilized in web development [20] and has been adapted in nf-test for Nextflow pipelines. Its naming and parameters are inspired by Jest [21]. nf-test captures a snapshot of output channels or other provided objects and compares them to reference snapshot files stored alongside the tests. Each snapshot file is a JSON file containing a serialized version of its content. When a file is added to a snapshot, its MD5 hash is stored instead of the file content. Additionally, nf-test can save the MD5 hash of an entire snapshot, enabling a compressed representation of large or complex content.

Because snapshot files are simple text files, they can be checked into version control systems and support human-readable diffs. During each test run, nf-test compares the actual snapshot with the reference snapshot. If the two snapshots differ, the test fail (see **Figure 2b**). If the change is unexpected, the user should address the bug detected by the test. Otherwise, the reference snapshot must be updated to reflect the new output of a process, workflow, pipeline, or function. In this case, the user runs nf-test with the "*--update-snapshot"* option.

All snapshot files are automatically included in the dependency graph, ensuring that tests are triggered whenever a snapshot changes. In addition, nf-test provides a CI mode (*--ci*), which prevents automatic snapshot updates and enforces test failures when discrepancies occur.

Extensions for bioinformatics

nf-test provides a plugin system for reusing code snippets, saving development time, simplifying test code and enhancing maintainability. The plugin system, built on Groovy, is well documented to encourage users to create and share their own extensions. For example, in most bioinformatics file formats, file determinism is not always guaranteed, as timestamps or input filenames can prevent files from being byte-identical. In such cases, MD5 hashs cannot be used for verification and implementing tests to validate the dynamic output can be time-consuming. To address this, nf-test provides plugins with built-in methods to extract and validate information from well-known bioinformatics file formats. Currently, nf-test includes plugins for VCF, BAM, FASTA, FASTQ, and CSV files (see [22]). For example, the nft-vcf plugin allows users to access file summaries, metadata, individual variants, and other key attributes, enabling flexible and automated testing. These plugins simplify writing nf-test assertions and make testing more convenient and powerful for end users.

Evaluation and Validation

We evaluated nf-test using three publicly available Nextflow pipelines to demonstrate its effectiveness in pipeline testing. While these examples focus on nf-core pipelines, nf-test is a general-purpose framework that can also be applied to other Nextflow-based projects outside the nf-core ecosystem. For instance, nf-test has been used with the publicly available genotype imputation pipeline employed by popular imputation servers [23]. We first assessed the effect of smart testing on optimizing test execution times and parallelization (see Results section "Optimizing Testing Efficiency and Performance"). This was done by simulating four specific changes through manual file modifications in the nf-core/fetchngs (Version 1.12.0, [24]) pipeline. We ran nf-test with the "*--related-tests*" option, which executes only tests relevant to the specified files. We also evaluated nf-core/modules (Commit ca199cf, [25]), a repository whose modules are already covered by nf-test tests, by running nf-test on the last 500 commits, using the "*--changed-since HEAD^*" flag to capture changes between consecutive versions. Second, to evaluate parallelization, we implemented 39 test cases and set up CI using GitHub Actions for the nf-gwas pipeline (Version 1.05, [18]). We tested it by employing test-list sharding across five machines with the option "*--shard i/5*", comparing both default and round-robin strategies for test case distribution. Speedup was measured as the ratio of execution times with and without test-list sharding. All analyses were conducted using nf-test 0.9.0 and results were visualized using R 4.3.3 and ggplot2 3.5.0.

RESULTS

Optimizing Testing Efficiency and Performance

Resource saving through smart testing

First, we evaluated smart testing and its impact on execution time using the nf-core/fetchngs pipeline. This pipeline includes 50 test cases for 17 components. The dependency graph illustrates the connections and dependencies between modules, workflows and test cases. Because each component includes at least one test case, the pipeline achieves 100% coverage (see **Figure S1**). The total execution time for all tests is 1,122 seconds. As hypothesized, we observed that pipeline end-to-end tests exhibit the slowest performance (see **Table 1**). We simulated various modifications to assess the impact of changes, varying the number of modified files and the type of change: (a) changes to the logic of the module itself and (b) changes to the module interface (e.g., adding a new input channel). Results show that smart testing saves between 46% and 80% of execution time by minimizing the number of executed test cases (see **Table 2**). We reviewed the results from a full run of the entire test suite and confirmed that all changes were detected by our approach.

Second, we analyzed the last 500 commits in the nf-core/modules project, spanning from October 26, 2023 to February 23, 2024. At the time of writing, nf-core/modules contains 1,150 modules, 56 workflows and more than 800 test cases implemented by the community. As expected, most commits and pull requests (PRs) affect only a single module. In such cases, time savings are significantly higher, as only the relevant unit tests and potential workflow integration tests need to be executed. nf-test accurately identified the specific test cases and integration tests required for the committed changes (see **Figure 4a**). Approximately 30% of the commits required executing more than 25 test cases, with most of these commits involving refactoring or restructuring tasks (see **Figure 4b**). The total number of executed test cases

was reduced from 238,205 to 1,600. nf-test parsed and analyzed 1,560 unique files in under one second to construct the dependency graph.

Execution time reduction through parallelization

We evaluated the efficiency and speedup of parallelization using nf-gwas [26], a pipeline for genome-wide association studies, which includes multiple long-running end-to-end tests (see **Figure S2** for the dependency graph). Initially, executing all 49 test cases on a single machine required 1,718 seconds. Execution time was reduced to 487 seconds by distributing the workload across five machines, achieving a speedup of 3.5. The default strategy distributes tests by name, which can result in unbalanced execution times, especially when outliers are present (see **Table 3**). Using a round-robin approach further reduced execution time to 333 seconds, resulting in a speedup of 5.2.

Code reduction through snapshot testing

We analyzed how snapshot testing in nf-test can improve quality and maintainability by reducing the effort required to write. Writing manual assertions for each output item can be time-consuming and error-prone, especially in pipelines with large numbers of outputs. For example, the nf-gwas pipeline generates five output files for a single phenotype. Writing a simple regression test requires creating an assertion for each file to verify its existence and another to ensure its content matches expectations. This manual process entails generating an MD5 hash for each file and using it in the corresponding assert block, resulting in 15 lines of code for three phenotypes. Additionally, the test case must be updated whenever the pipeline generates additional output files, requiring manual synchronization. Snapshot testing streamlines this process by replacing 15 lines of code with a single line. nf-test automatically creates the MD5 hash for each file on the first run and provides commands to update the reference snapshot.

We adapted the nf-gwas pipeline and simulated various software updates for REGENIE [27], the underlying software: (a) changes in the output file format (renaming the column “LOG10P” to “PVALUE”), (b) a bug in association detection (where six variants are no longer genome-wide significant), and (c) modifications to default parameters. Change (a) breaks the pipeline and is easy to detect without test cases by running the pipeline on test data. However, changes (b) and (c) produce incorrect output results without breaking the pipeline. While these scenarios are particularly challenging for a pipeline developer, parameter or result changes can also outputs that are still valid, and in some cases, even more biologically meaningful. The implemented test cases detected all of these issues. For example, the unit test for REGENIE, which checks whether 116 variants are genome-wide significant, fails because only 110 variants were found. Similarly, changes in default parameters (e.g., a more restricted MAF filter) produce a different number of resulting variants, causing the test to fail.

Comparison to other frameworks

nf-test is a flexible testing framework for Nextflow pipelines, going beyond existing solutions such as NFTest and pytest-workflow (see **Table 4**)**.** Unlike these systems, nf-test integrates unit, snapshot, and end-to-end testing into a single extendable DSL, while also supporting advanced features like optimized test strategies, dependency analysis, coverage reporting, and parallelized execution. It introduces bioinformatics-aware assertions (e.g., for VCF, BAM or FASTA files), modular and portable test design, and multiple output formats (JUnit, XML, TAP, CSV), making it highly suitable for large-scale scientific workflows. In contrast, NFTest and pytest-workflow provide more limited functionality, requiring manual setup for unit or snapshot testing and lacking support for advanced optimizations, dependency tracking, or domain-specific assertions. nf-test combines flexibility, reproducibility, and portability in a way that existing tools do not. nf-test has established itself as the tool of choice within the Nextflow and nf-core communities, which provide best-practice pipelines for a wide range of use cases.

DISCUSSION

Since its first release in October 2021, nf-test has been integrated into dozens of pipelines and downloaded over 200,000 times. As the new standard testing framework in nf-core for both pipelines and provided modules, it shows a high level of acceptance, reflecting the community's recognition of the importance of testing pipelines.

Various efforts have previously addressed Nextflow pipeline testing, including NFTest [13] and the nf-core framework [3], which utilize pytest-workflow [28]. However, these solutions rely on on YAML files with predefined assertions, are limited to end-to-end testing, depend on external scripts to validate output files and do not support optimization strategies such as parallelization and smart testing. nf-test addresses these limitations by providing a dedicated testing framework that simplifies writing, executing, and analyzing tests for Nextflow pipelines. It offers a DSL that follows the naming conventions and philosophy of Nextflow DSL 2 and enables writing complex assertions, which are often required to validate the extensive outputs of bioinformatics analysis. Because the DSL is based on Groovy, users can extend it and leverage the rich ecosystem of Java/JVM libraries in bioinformatics. Additionally, sharing domain-specific assertions via plugins facilitates collaboration among users.

We implemented a unit testing approach in which all components of a Nextflow pipeline can be tested without manually writing additional Nextflow workflows to execute a subprocess with test data. This modularity also facilitates writing integration tests for sub-workflows, ensuring that processes interact as expected. Testing is conducted consistently across a project, allowing side effects to be detected early. The modular design simplifies debugging, encourages iterative development, and promotes code reuse. Each module can be individually tested and seamlessly integrated into the final pipeline or workflow composition. Integration tests are particularly important when using large module libraries, such as those provided by nf-core/modules, to ensure that third-party updates do not break pipelines

In pipelines with extensive outputs, manually writing assertions for each output item can be complex. To address this, nf-test introduces snapshot testing. Instead of specifying individual assertions for each output, snapshots capture the state of the output channels or folders, including file names and hash values. These snapshots are automatically generated during the first run, and nf-test compares the current output with the reference snapshot in subsequent runs. This approach streamlines regression testing, ensures reproducibility, and helps catch regressions early in development. Snapshot testing in nf-test is particularly useful in two scenarios: First, it supports pipeline and module refactoring, where outputs are expected to remain unchanged, ensuring that modifications to the Nextflow logic do not alter the results. Second, it aids version management, as test failures can indicate changes in underlying tools or dependencies, signaling that a new version or review may be required. Outside of these cases, traditional testing approaches are generally more appropriate. nf-test therefore also provides mechanisms for assertion-based tests, allowing verification of file or channel contents, tool execution checks to confirm expected behavior for given inputs, and error-handling tests that ensure robust failure responses. Together, these testing strategies provide a balanced framework that combines the change-detection benefits of snapshot testing with the precision of conventional verification methods. Evaluation of the nf-gwas pipeline demonstrated that nf-test and snapshot testing improve code quality and maintainability while reducing the effort required to write manual assertions.

Regression testing involves retesting pipelines after any code modification. Given that bioinformatics pipelines process large input data and employ complex algorithms, full regression tests can take hours. To save effort and time, nf-test only reruns tests affected by a modification. Using the nf-core/modules project as an example, we showed that most changes and commits in such large projects affect only a specific set of modules and tests. Therefore, nf-test implements strategies to identify the minimal set of tests that must be rerun. This results in resource savings and faster development cycles, up to an 80% reduction in execution time.

When pipelines contain large numbers of tests, test-list sharding can split tests across multiple machines. Experimental results showed performance gains of up to 80% using five machines. However, there is no guarantee of an optimal or fair split among resources as the splitting decision is not influenced by data from previous runs. The implemented round-robin strategy simply attempts to distribute the workload evenly. Nonetheless, the setup remains easy since no shared database, queuing system or orchestration instance is needed. Combined with the integration of Git, nf-test enables the setup of Test-Driven Development (TDD) and CI for Nextflow pipelines.

The implemented dependency analysis provides an overview of pipeline coverage and quantifies the testing effort. The current implementation has limitations, as it only indicates whether at least one test case exists per unit, without reflecting whether all instructions or branches are covered. Future work aims to extend this approach and to include metrics that reflect the complexity of change sets. Additionally, nf-test currently depends on local environments, which can limit adaptability to certain infrastructures. For example, running test cases across different cloud providers is currently limited and will be addressed in future versions.

Availability of Source Code and Requirements

Project name: nf-test

Project home page: https://github.com/askimed/nf-test

Operating system(s): Platform independent

Programming language: Java

License: MIT

RRID: SCR_026663

DATA AVAILABILITY

nf-test and its documentation are available at website [29] and Bioconda [30]. Associated plugins is available at [31].

ACKNOWLEDGMENTS

We would like to express our gratitude to the nf-core community for their support in this project, including their contributions of test cases and valuable suggestions. Special thanks go to Sateesh Peri, Maxime Garcia, Edmund Miller, Nicolas Vannieuwkerke, Harshil Patel, Adam Talbot, and all GitHub contributors.

AUTHOR CONTRIBUTIONS

LF conceptualized and supervised the project. LF and SS implemented the software. LF and SS conducted formal analyses and wrote the manuscript (original draft, review and editing).

CONFLICT OF INTEREST

None declared.

FUNDING STATEMENT

This work has been supported by the Medical University of Innsbruck.

FIGURES

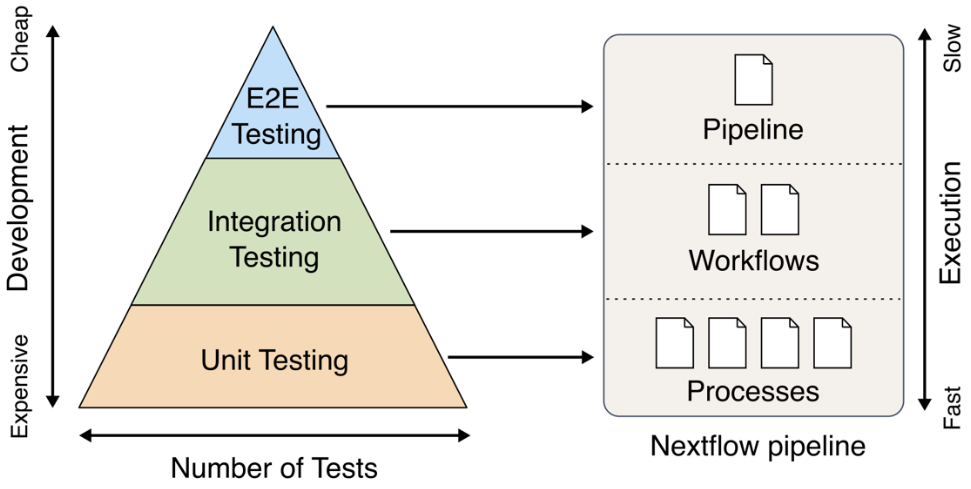

Figure 1: Overview of different test strategies provided by nf-test. A comprehensive and efficient test strategy for a Nextflow project includes unit, integration, and end-to-end testing. Unit tests are inexpensive to execute but require more effort to develop. In contrast, integration and end-to-end tests are easier to write but more costly to run, highlighting the trade-off between development effort and execution time across testing levels.

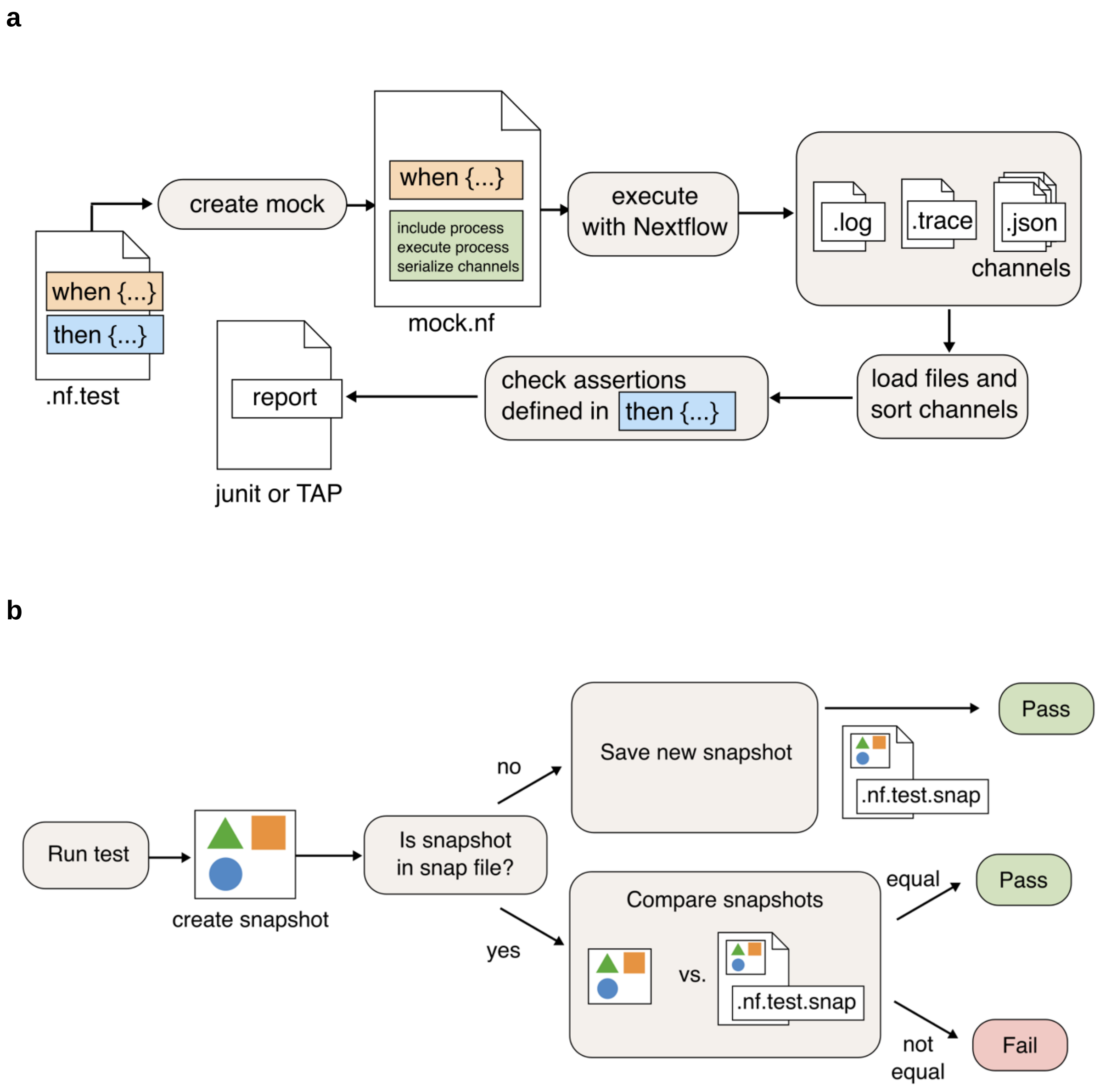

Figure 2: Architecture of the implemented test framework. (a) nf-test generates Nextflow scripts for tests: (i) initializes Nextflow unit with “when” block parameters, (ii) executes the unit and serializes output channels, (iii) parses channel content, evaluates “then” block assertions for output validation. Test results are aggregated and reported in multiple formats. **(b)** nf-test runs a test, creates and compares output objects against reference snapshot files stored with the tests. A test fails if the snapshots don't match, indicating either unexpected changes or the need to update the reference snapshot to reflect new outputs.

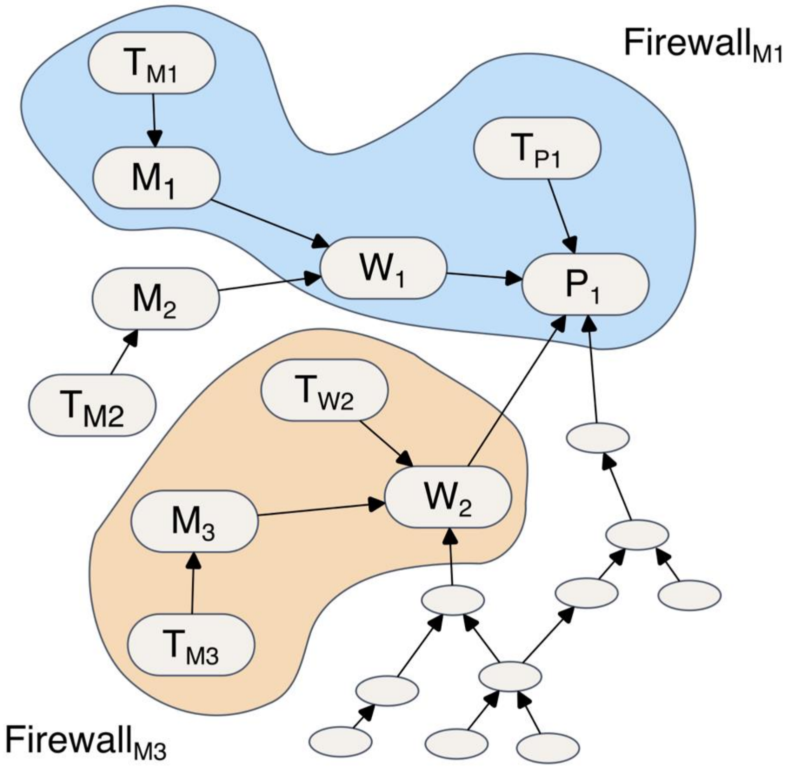

Figure 3: Example of a dependency graph and firewalls. The figure illustrates the dependencies of tests (T_x), modules/processes (M_x), workflows (W_x), and pipelines (P_x) within a Nextflow project. Changes to module M_1 will only affect test cases inside Firewall M_1. Firewall M_3 is more compact because workflow W_2 contains a test case ensuring the integrity of M_3. This approach avoids running expensive end-to-end tests for P_1.

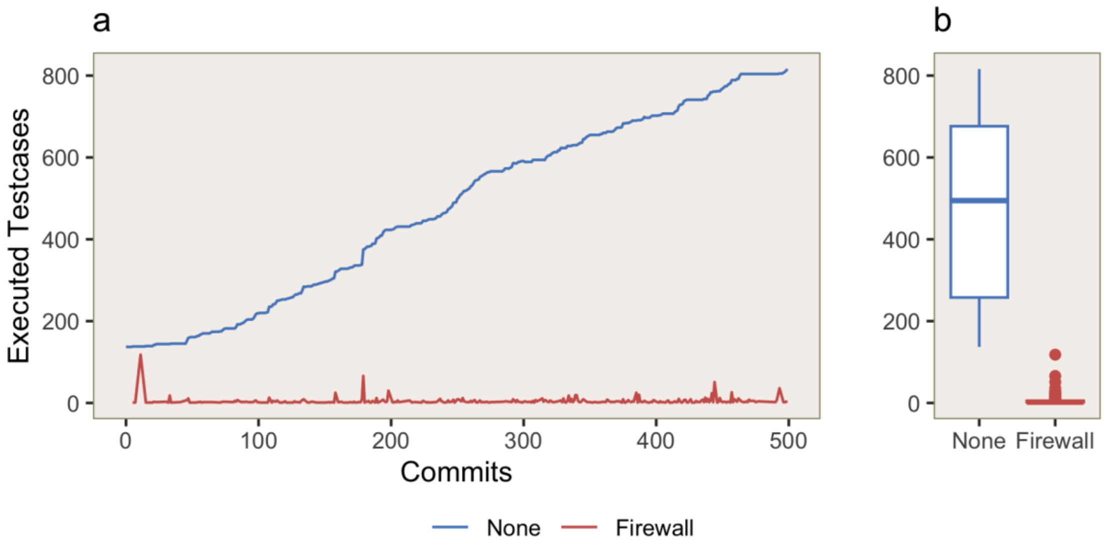

Figure 4: Last 500 commits of the nf-core/modules projects between 2023/10/26 and 2024/02/23. (A) The blue line represents the number of test cases that would be executed without any optimization strategy. The red line depicts the number of executed test cases when using the implemented Firewall strategy. (B) Boxplot of the number of executed test cases per commit.

TABLES

Table 1: Number of test cases in the nf-core/fetchngs pipeline (Version 1.12.0). Each of the 17 components has at least one test case, for a total of 50 test cases. The total execution time is 1,122 sec.

			Execution Time	
	Tests suites	Tests cases	Mean (sec)	Total (sec)
Functions	3	14	2.6	36.5
Pipelines	1	1	512.1	512.1
Modules/Processes	10	13	11.2	145.7
Workflows	15	22	19.4	427.8
Total	29	50	-	1,122.0

Table 2: Time and resource saving for different modifications of the nf-core/fetchngs. We simulated several typical modifications and measured the execution time using nf-test's optimization strategy. Time savings are calculated based on the execution time of a full run (1,122 sec).

		Execution Time		
Modification	Executed test cases	Mean (sec)	Total (sec)	Saving
changed module sra_to_samplesheet	10	22.3	222.5	80.2%
changed modules sra_to_samplesheet multiqc_mappings_config	11	52.7	579.4	48.4%
update of a nf-core module: utils_nfcore_pipeline	23	14.4	332.1	70.4%
changed main workflow main.nf	1	596.3	596.3	46.8%

Table 3: Time and speedup for different sharding strategies of the nf-gwas pipeline.

Strategy	Shards	Median (sec)	Total Time	Speed Up
none	5	368 +/-164	487 sec	3.52
Round-Robin	5	314 +/- 10	333 sec	5.15

Table 4: Comparison of nf-test with similar approaches.

	nf-test	NFTest	pytest-workflow
**End to End Testing**	Yes	Yes	Yes
**Unit Testing**	Yes	Manual writing Nextflow script	Manual writing Nextflow script
**Snapshot Testing**	Yes	Manual preparation of expected output files	Manual preparation of expected output files
**Optimized test strategies**	Yes	No	No
**Dependency analysis**	Yes	No	No
**Coverage reporting**	Yes	No	No
**Tags**	Yes	No	Yes
**Output formats**	Junit, xml, TAP and csv	No	Junit, html
**Code generation**	config file and for each unit	config file	Using nf-core tools
**Custom assertions**	Yes	Through external third party scripts	No
**Bioinformatics support**	Yes (e.g. vcf, fasta, …)	No	No
**Parallelization**	Sharding	No	No
**Modularity and portability**	Each unit has its own test files that can be transferred	One test file for whole project	Multiple files
**Writing test cases**	Extendable DSL	YAML with predefined structure	YAML with predefined structure
**License**	MIT	GPL-2.0	AGPL-3.0
**Website**	https://www.nf-test.com	https://github.com/uclahs-cds/tool-NFTest	https://pytest-workflow.readthedocs.io

REFERENCES

1. Kumar P, Paul RK, Roy HS, Yeasin M, Ajit, Paul AK: **Big Data Analysis in Computational Biology and Bioinformatics**. *Methods Mol Biol* 2024, **2719**:181-197. https://doi.org/10.1007/978-1-0716-3461-5_11.
2. Di Tommaso P, Chatzou M, Floden EW, Barja PP, Palumbo E, Notredame C: **Nextflow enables reproducible computational workflows**. *Nat Biotechnol* 2017, **35**(4):316-319. https://doi.org/10.1038/nbt.3820.
3. Ewels PA, Peltzer A, Fillinger S, Patel H, Alneberg J, Wilm A, Garcia MU, Di Tommaso P, Nahnsen S: **The nf-core framework for community-curated bioinformatics pipelines**. *Nat Biotechnol* 2020, **38**(3):276-278. https://doi.org/10.1038/s41587-020-0439-x.
4. Langer BE, Amaral A, Baudement M-O, Bonath F, Charles M, Chitneedi PK, Clark EL, Di Tommaso P, Djebali S, Ewels PA *et al*: **Empowering bioinformatics communities with Nextflow and nf-core**. *Genome Biology* 2025, **26**(1):228. https://doi.org/10.1186/s13059-025-03673-9.
5. Electrical Io, Engineers E: **Software Engineering Standards: ANSI/IEEE Std 729-1983, Glossary of Software Engineering Terminology**: Inst. of Electrical and Electronics Engineers; 1984.
6. Piras ME, Pireddu L, Zanetti G: **wft4galaxy: a workflow testing tool for galaxy**. *Bioinformatics* 2017, **33**(23):3805-3807. https://doi.org/10.1093/bioinformatics/btx461.
7. Krafczyk M, Shi A, Bhaskar A, Marinov D, Stodden V: **Scientific Tests and Continuous Integration Strategies to Enhance Reproducibility in the Scientific Software Context**. In: *Proceedings of the 2nd International Workshop on Practical Reproducible Evaluation of Computer Systems: 2019; Phoenix, AZ, USA*. Association for Computing Machinery: 23–28. https://doi.org/10.1145/3322790.3330595.
8. Beizer B: **Software testing techniques (2nd ed.)**: Van Nostrand Reinhold Co.; 1990.
9. Kamali AH, Giannoulatou E, Chen TY, Charleston MA, McEwan AL, Ho JWK: **How to test bioinformatics software?** *Biophys Rev* 2015, **7**(3):343-352. https://doi.org/10.1007/s12551-015-0177-3.
10. Ferenc K, Rauluseviciute I, Hovan L, Kumar V, Kuijjer ML, Mathelier A: **Improving bioinformatics software quality through teamwork**. *Bioinformatics* 2024, **40**(11). https://doi.org/10.1093/bioinformatics/btae632.
11. Djaffardjy M, Marchment G, Sebe C, Blanchet R, Bellajhame K, Gaignard A, Lemoine F, Cohen-Boulakia S: **Developing and reusing bioinformatics data analysis pipelines using scientific workflow systems**. *Comput Struct Biotechnol J* 2023, **21**:2075-2085. https://doi.org/10.1016/j.csbj.2023.03.003.
12. van der Putten BCL, Mendes CI, Talbot BM, de Korne-Elenbaas J, Mamede R, Vila-Cerqueira P, Coelho LP, Gulvik CA, Katz LS, The Asm Ngs Hackathon P: **Software testing in microbial bioinformatics: a call to action**. *Microb Genom* 2022, **8**(3). https://doi.org/10.1099/mgen.0.000790.
13. Patel Y, Zhu C, Yamaguchi TN, Bugh YZ, Tian M, Holmes A, Fitz-Gibbon ST, Boutros PC: **NFTest: automated testing of Nextflow pipelines**. *Bioinformatics* 2024, **40**(2). https://doi.org/10.1093/bioinformatics/btae081.
14. **nf-test website and documentation.** https://www.nf-test.com. [accessed 2025 Oct 19]

15. **Test Anything Protocol (TAP).** http://testanything.org. [accessed 2025 Oct 19]

16. White LJ, Leung HK: **A firewall concept for both control-flow and data-flow in regression integration testing**. In: *Proceedings Conference on Software Maintenance: 1992*. IEEE Computer Society: 262-271. https://doi.org/10.1109/ICSM.1992.242535.

17. Leung HK, White L: **A study of integration testing and software regression at the integration level**. In: *Proceedings Conference on Software Maintenance: 1990*. IEEE Computer Society: 290-301. https://doi.org/10.1109/ICSM.1990.131377.

18. **Git website.** https://git-scm.com. [accessed 2025 Oct 19]

19. Gonsalves M, Mandala J: **Regression Test List Sharding in a Distributed Test Environment**. In: *E3S Web of Conferences: 2023*. EDP Sciences: 04028. https://doi.org/10.1051/e3sconf/202339904028.

20. Gazzinelli Cruz VP, Rocha H, Valente MT: **Snapshot testing in practice: Benefits and drawbacks**. *Journal of Systems and Software* 2023, **204**:111797. https://doi.org/10.1016/j.jss.2023.111797.

21. **Jest is a delightful JavaScript Testing Framework.** https://jestjs.io. [accessed 2025 Oct 19]

22. **nf-test plugin repository.** https://plugins.nf-test.com. [accessed 2025 Oct 19]

23. Forer L, Taliun D, LeFaive J, Smith AV, Boughton AP, Coassin S, Lamina C, Kronenberg F, Fuchsberger C, Schonherr S: **Imputation Server PGS: an automated approach to calculate polygenic risk scores on imputation servers**. *Nucleic Acids Res* 2024, **52**(W1):W70-W77. https://doi.org/10.1093/nar/gkae331.

24. **nf-core/fetchngs: a bioinformatics pipeline to fetch metadata and raw FastQ files.** https://github.com/nf-core/fetchngs. [accessed 2025 Oct 19]

25. **nf-core/modules: a repository for hosting Nextflow DSL2 module files** https://github.com/nf-core/modules. [accessed 2025 Oct 19]

26. Schonherr S, Schachtl-Riess JF, Di Maio S, Filosi M, Mark M, Lamina C, Fuchsberger C, Kronenberg F, Forer L: **Performing highly parallelized and reproducible GWAS analysis on biobank-scale data**. *NAR Genom Bioinform* 2024, **6**(1):lqae015. https://doi.org/10.1093/nargab/lqae015.

27. Mbatchou J, Barnard L, Backman J, Marcketta A, Kosmicki JA, Ziyatdinov A, Benner C, O'Dushlaine C, Barber M, Boutkov B *et al*: **Computationally efficient whole-genome regression for quantitative and binary traits**. *Nat Genet* 2021, **53**(7):1097-1103. https://doi.org/10.1038/s41588-021-00870-7.

28. **pytest-workflow documentation.** https://pytest-workflow.readthedocs.io/. [accessed 2025 Oct 19]

29. nf-test: A simple testing framework for Nextflow pipelines. https://www.nf-test.com. Accessed March 24, 2025.

30. Bioconda: https://bioconda.github.io/recipes/nf-test/README.html. Accessed March 24, 2025.

31. Explore nf-test plugins made by the community. https://plugins.nf-test.com/. Accessed March 24, 2025.

figure 1

Click here to access/download;Figure;Fig 1.png

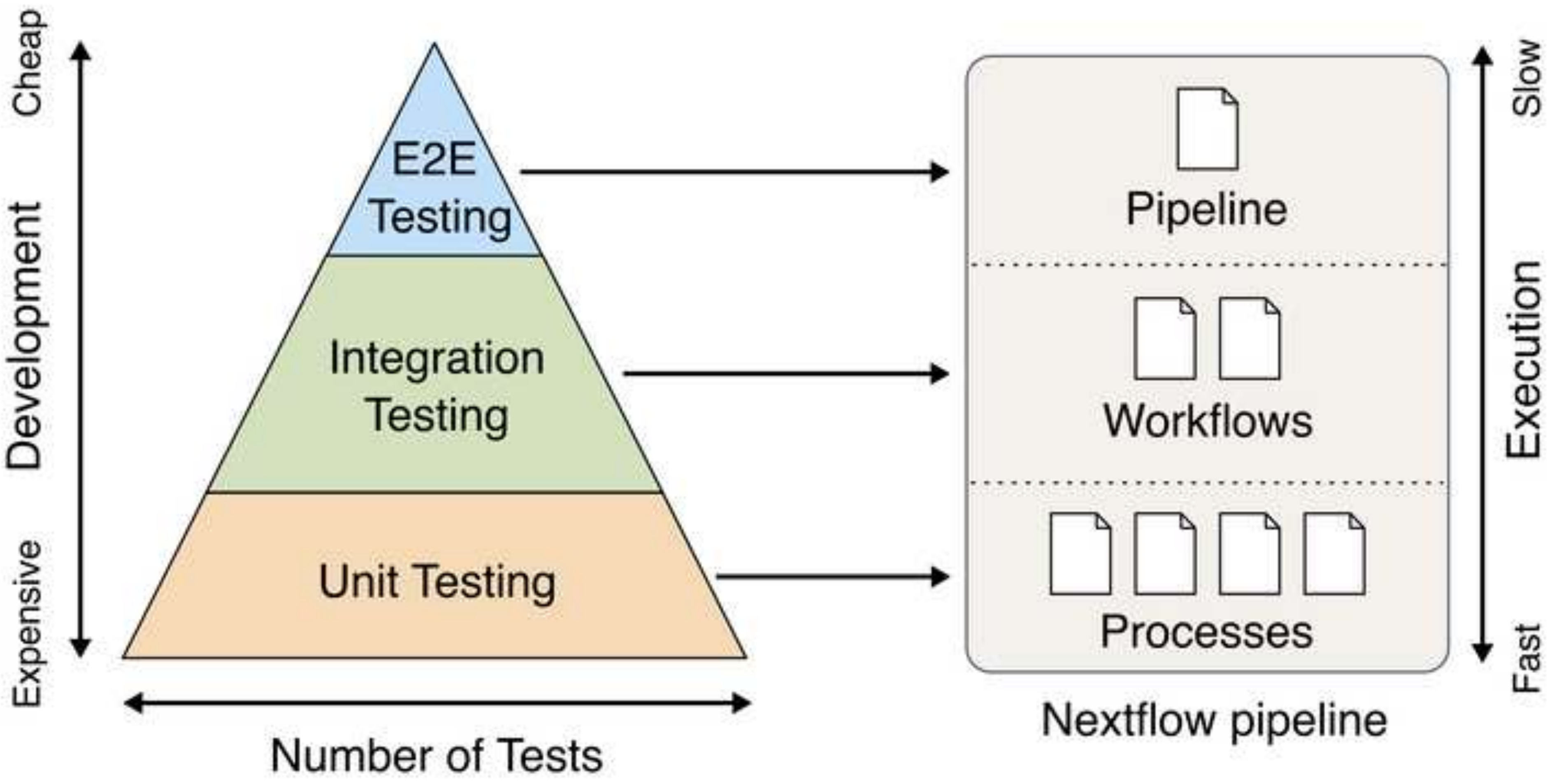

figure 2

Click here to access/download;Figure;Fig 2.png

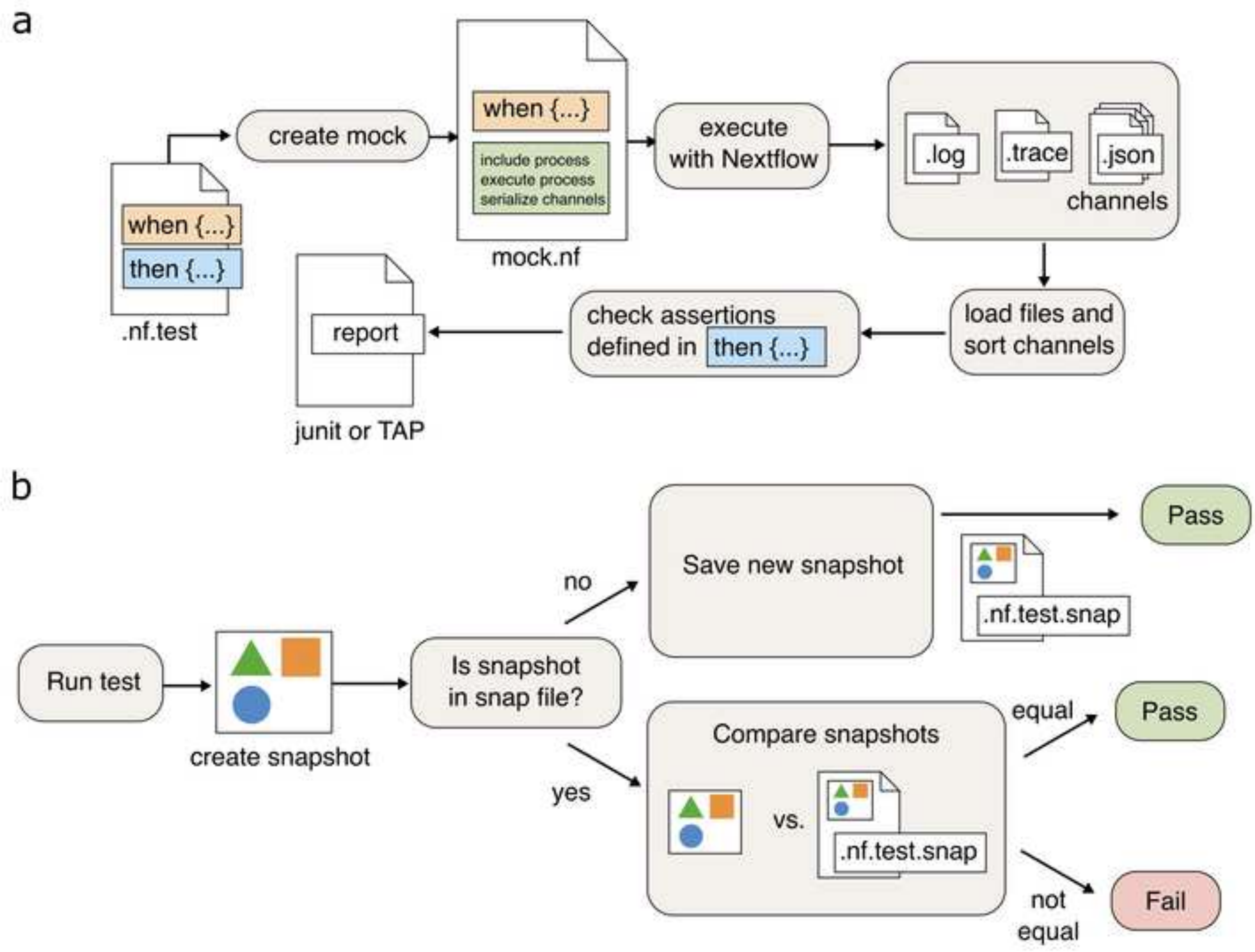

figure 3

Click here to access/download;Figure;Fig 3.png

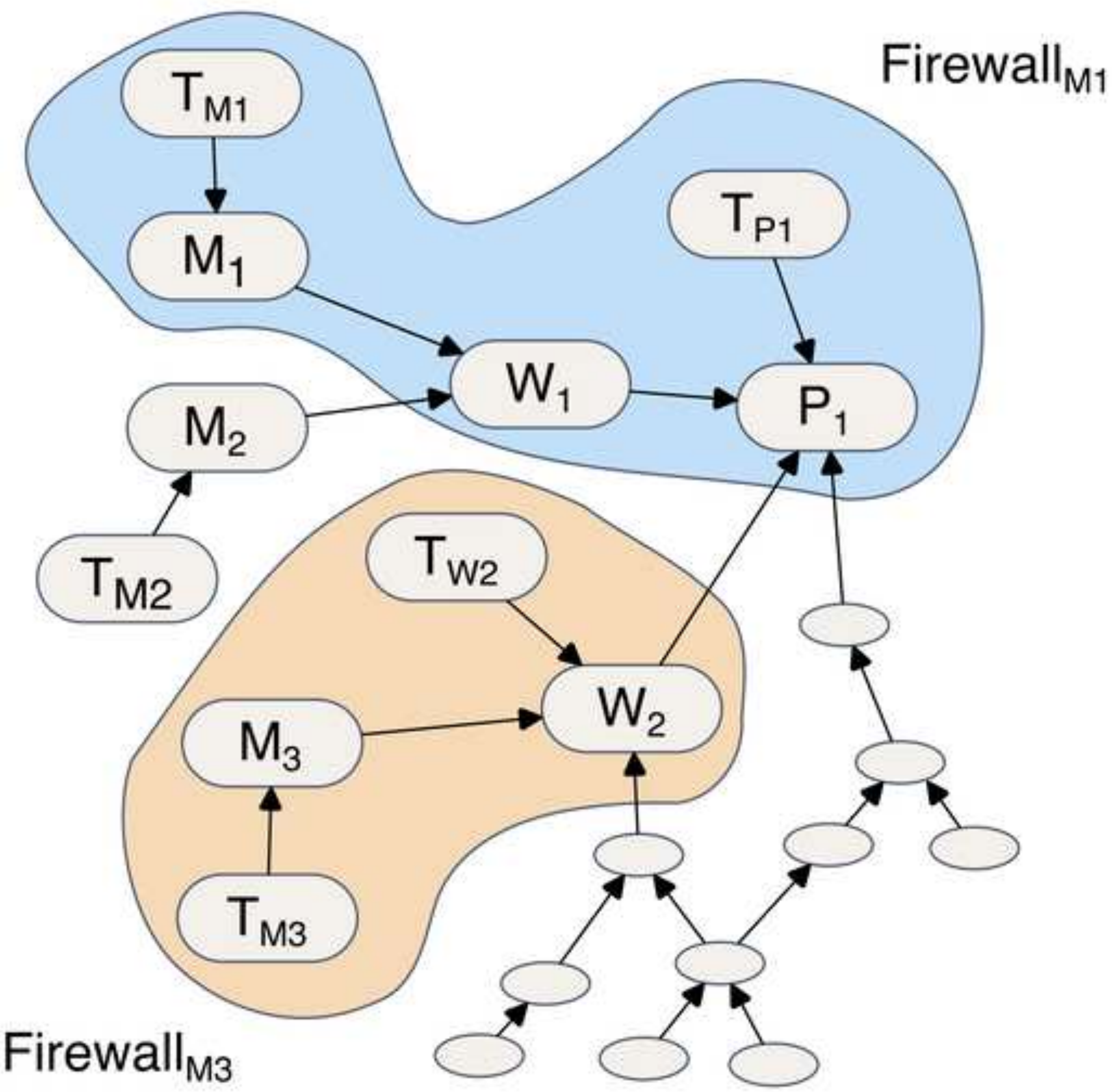

figure 4

Click here to access/download;Figure;Fig 4.png

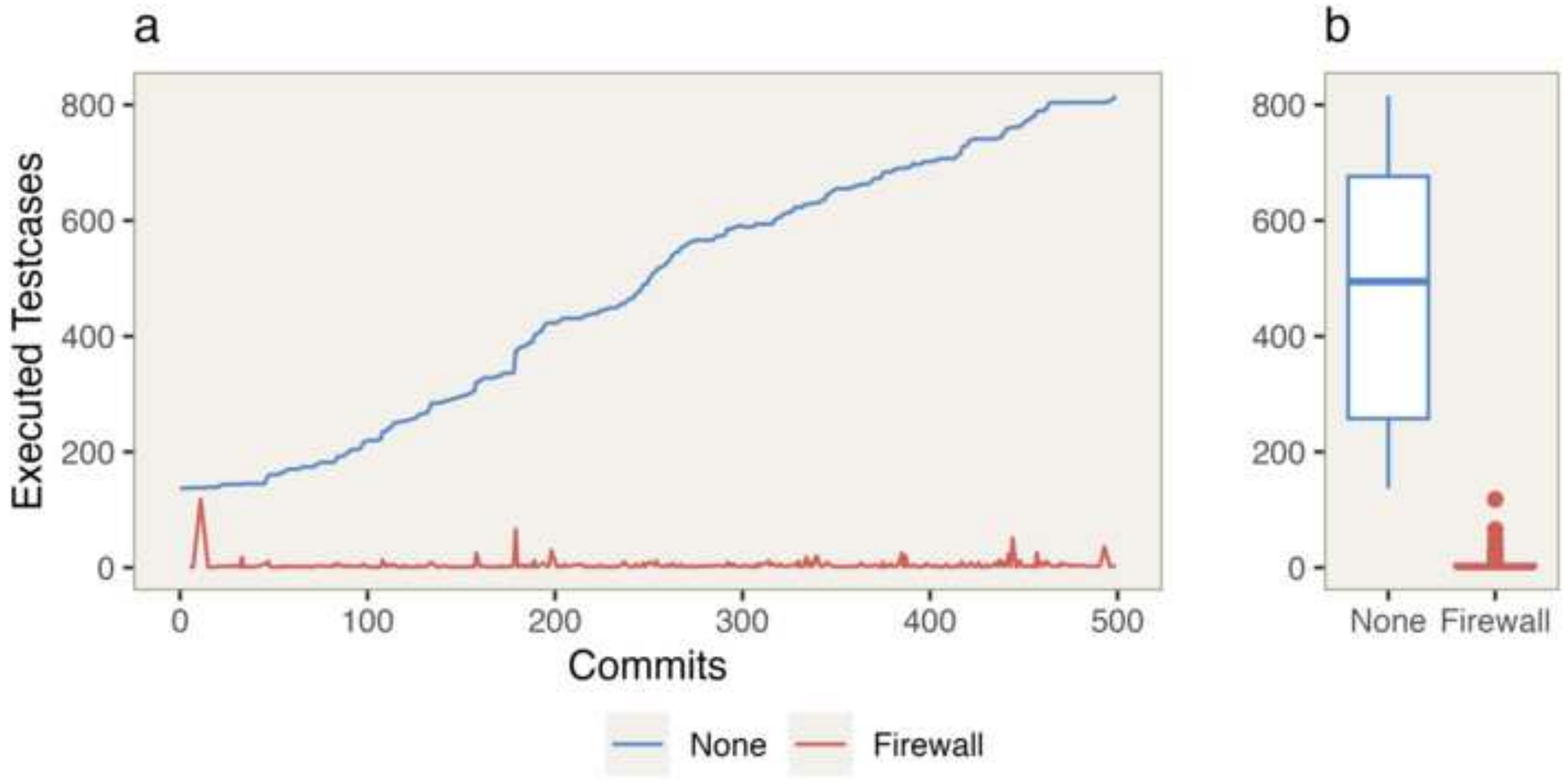

Click here to access/download
Supplementary Material
supplemental-material.pdf

Click here to access/download
Supplementary Material
revision-letter.pdf
