# Supplementary material for: Improving the reliability, quality, and maintainability of bioinformatics pipelines with nf-test
Source: Gigascience. 2025 Oct 22;14:giaf130. doi: 10.1093/gigascience/giaf130 (PMC12616847; doi:10.1093/gigascience/giaf130)
Supplement: giaf130_Reviewer_1_Report_Original_Submission — Jose Espinosa-Carrasco -- 5/19/2025 [file giaf130_reviewer_1_report_original_submission.pdf]

Reviewer Report

Title: Improving the Reliability, Quality and Maintainability of Bioinformatics Pipelines with nf-test

Version: Original Submission **Date:** 5/19/2025

Reviewer name: Jose Espinosa-Carrasco

Reviewer Comments to Author:

The article presents nf-test, a new modular and automated testing framework designed specifically for Nextflow workflows, a widely used workflow management system in bioinformatics. nf-test aims to help developers improve the reliability and maintainability of complex Nextflow pipelines. The framework includes very useful features such as snapshot testing, which assesses the computational repeatability of the results produced by the execution of a pipeline or its components and smart testing which optimises computational resources by only executing tests on the parts of the pipeline that were modified, reducing overall run time. Notably, nf-test can be integrated into CI workflows and has already been adopted by the nf-core community, demonstrating its utility and maturity in real-world scenarios

General comments:

The manuscript could benefit from reordering some sections to follow a more consistent structure and by removing redundant explanations.

I think it would be nice to include one limitation of nf-test, the fact that reproducing previous results does not necessarily imply biological correctness. This point is not entirely clear in the current version of the manuscript (see my comment below).

Another aspect that could improve the manuscript is the inclusion of at least one reference or explanation of how nf-test can be applied outside nf-core pipelines, as all the provided examples are currently restricted to nf-core.

Specific comments:

On page 3, the sentence "Thus, maintenance requires substantial time and effort to manually verify that the pipeline continues to produce scientifically valid results" could be more precise. I would argue that identical results across versions do not guarantee scientific validity; they merely confirm consistency with previous outputs. True scientific validity requires comparison against a known ground truth or standard.

On page 4, in the sentence "It is freely available, and extensive documentation is provided on the website", I think it would be nice to include the link to the documentation.

In the "Evaluation and Validation" section (page 8), it would be helpful to briefly state the goal of each evaluated test, as is done with the nf-gwas example. ou could include something similar for the nf-core/fetchngs and modules examples (e.g. to assess resource optimization through smart testing). Also, the paragraph references the "--related-tests" option, which could benefit from a short explanation of what it does. Lastly, the order in which the pipelines are presented in this section differs from the order in the Results, which makes the structure a bit confusing.

The sections titled "Unit testing in nf-test", "Test case execution", "Smart testing and parallelization", "Snapshot testing", and "Extensions for bioinformatics" seem more appropriate for the Materials and

Methods section, as they describe the design and functionality of nf-test rather than reporting actual results. Please ignore this comment if the current structure follows specific journal formatting requirements that I may not be aware of.
The Snapshot testing discussion in the Results section feels somewhat repetitive with its earlier explanation. Consider combining both discussions or restructuring the content to reduce duplication.
On page 11, the sentence "In these cases, MD5 sums cannot be used and validating the dynamic output content can be time-intensive" is not entirely clear to me, does it mean that it is time consuming to implement the test for this kind of files or that the validation of the files is time consuming?
On page 12, the sentence "Second, we analyzed the last 500 commits..." is confusing because this is actually the third point in the "Evaluation and Validation" section, as mentioned before. reordering would improve clarity.
On page 14, the authors state "However, changes (b) and (c) lead to incorrect output results without breaking the pipeline. Thus, these are the worst-case scenarios for a pipeline developer." While this is mostly true, I would also add that a change in parameters may produce different, but not necessarily incorrect, resultsâ€”some may even be more biologically meaningful. I suggest to acknowledge this.
Typos:
In the abstract: "Build on a similar syntax as Nextflow DSL2" should be corrected to "Built on a similar syntax as Nextflow DSL2".
In the legend of Figure 2 (page 19): "nf-tet" should be "nf-test".
In the legend of Table 2: "Time savings areis calculated..." should be "Time savings are calculated..."
Recommendation:
Given the relevance and technical contributions of the manuscript, I recommend its publication after addressing the minor revisions summarized above.

Methods

Are the methods appropriate to the aims of the study, are they well described, and are necessary controls included? Choose an item.

Conclusions

Are the conclusions adequately supported by the data shown? Choose an item.

Reporting Standards

Does the manuscript adhere to the journal's guidelines on minimum standards of reporting? Choose an item.

Choose an item.

Statistics

Are you able to assess all statistics in the manuscript, including the appropriateness of statistical tests used? Choose an item.

Quality of Written English

Please indicate the quality of language in the manuscript: Choose an item.

Declaration of Competing Interests

Please complete a declaration of competing interests, considering the following questions:

- Have you in the past five years received reimbursements, fees, funding, or salary from an organisation that may in any way gain or lose financially from the publication of this manuscript, either now or in the future?
- Do you hold any stocks or shares in an organisation that may in any way gain or lose financially from the publication of this manuscript, either now or in the future?
- Do you hold or are you currently applying for any patents relating to the content of the manuscript?
- Have you received reimbursements, fees, funding, or salary from an organization that holds or has applied for patents relating to the content of the manuscript?
- Do you have any other financial competing interests?
- Do you have any non-financial competing interests in relation to this paper?

If you can answer no to all of the above, write 'I declare that I have no competing interests' below. If your reply is yes to any, please give details below.

I declare that I have no competing interests

I agree to the open peer review policy of the journal. I understand that my name will be included on my report to the authors and, if the manuscript is accepted for publication, my named report including any attachments I upload will be posted on the website along with the authors' responses. I agree for my report to be made available under an Open Access Creative Commons CC-BY license (http://creativecommons.org/licenses/by/4.0/). I understand that any comments which I do not wish to be included in my named report can be included as confidential comments to the editors, which will not be published.

Choose an item.

To further support our reviewers, we have joined with Publons, where you can gain additional credit to further highlight your hard work (see: https://publons.com/journal/530/gigascience). On publication of this paper, your review will be automatically added to Publons, you can then choose whether or not to claim your Publons credit. I understand this statement.

Yes Choose an item.
