# Supplementary material for: Improving the reliability, quality, and maintainability of bioinformatics pipelines with nf-test
Source: Gigascience. 2025 Oct 22;14:giaf130. doi: 10.1093/gigascience/giaf130 (PMC12616847; doi:10.1093/gigascience/giaf130)
Supplement: giaf130_Reviewer_2_Report_Original_Submission — Katalin Ferenc -- 7/1/2025 [file giaf130_reviewer_2_report_original_submission.pdf]

Reviewer Report

Title: Improving the Reliability, Quality and Maintainability of Bioinformatics Pipelines with nf-test

Version: Original Submission **Date:** 7/1/2025

Reviewer name: Katalin Ferenc

Reviewer Comments to Author:

1) General assessment of the work.
It is a very nice addition to the scientific community, an important step towards standardizing the development and maintenance of software for bioinformatics pipelines. It is not a trivial task to adapt unit testing concepts to pipelines. nf-test has already been used by the community and has been in a feedback loop with the users. Thus, its usability has been constantly improving, both through the efforts of the developers and additional plugins from the user base, highlighting the ease of contribution to the nf-test software base. The text is well written and easy to follow. However, some concepts could be better described and discussed for the readers.
2) Specific comments for revision:
a) Major comments;
- The authors should refer to pytest-workflow in the introduction, along with NFTest, as both are used for comparison.
- Test coverage is helpful to identify which lines are vulnerable to changes. For the calculation of the test coverage in nf-test, indirect tests are considered. Does it mean that if a single integration test is written, then all called modules are considered covered? Please clarify or argue why this is a good strategy.
- An interesting idea in nf-test is to use snapshot testing for modules, workflows, and pipelines. As the authors mention, this has been used in web development. According to the cited reference, it is especially used for frontend code and has been noted as a quick but fragile way of testing. This is because snapshot testing does not provide insight into the correctness of the code, but only asserts that there was no change. It is beneficial that this test checks for unexpected changes that unit tests might miss.
In the "Code reduction through snapshot testing" section, the authors highlight cases when snapshot testing results in failed tests: 1) when there is a change in the code due to a bug, and 2) when default parameters are modified. We understand that snapshot testing in the context of pipeline development is useful in two scenarios:
1. when the pipeline itself is being refactored, the output of each module should stay the same. In this case, snapshot testing is used to fix the output of the tools, and a failing test highlights that the Nextflow code wrapping the tools is incorrectly integrated (i.e., connected to each other).
2. pipeline / module versioning requires knowledge about changes in the underlying tools. In this case, snapshot testing helps because any failure in the tests flags a change. As there is no oracle, one would not know if the bug was introduced or fixed. However, from the pipeline development perspective, the only thing that matters is that there should be a new version.
According to our understanding, in any other case, a more traditional approach should be preferred,

where there is an oracle knowing about expected file formats, content, or errors. Otherwise, there is a risk of adding many tests that unnecessarily fail, causing increased development time. Please add explicit discussion about these scenarios, or other ones based on your insights, highlighting when snapshot testing is applicable/appropriate during pipeline development. Please add a summary of other types of tests (e.g., assertions about file or channel content, verification of tool execution given input data, and error handling checks) that can be run within the nf-test framework.
b) Minor comments:
- In the "evaluation and validation" section, the authors describe that they ran tests in nf-core/modules between github versions. Please clarify that these modules were already covered by tests.
- Table 4 is referenced in the Discussion section. It would be better to move the comparison between tools to the Results section.
- On page 16, typo: "queuing system"
- Figure 2 title typo: "nf-tet"
- Figure 2: please add comments about the time cost of adding tests during the development, as it is highlighted on the figure.
- Page 22 typo: "savings areis calculated"
- Abstract: "Build on…" should be "Built on…"
- Shouldn't TM2 linked to M3 be TM3 in Figure 1?

Methods

Are the methods appropriate to the aims of the study, are they well described, and are necessary controls included? Choose an item.

Conclusions

Are the conclusions adequately supported by the data shown? Choose an item.

Reporting Standards

Does the manuscript adhere to the journal's guidelines on [minimum standards of reporting?]() Choose an item.

Choose an item.

Statistics

Are you able to assess all statistics in the manuscript, including the appropriateness of statistical tests used? Choose an item.

Quality of Written English

Please indicate the quality of language in the manuscript: Choose an item.

Declaration of Competing Interests

Please complete a declaration of competing interests, considering the following questions:

- Have you in the past five years received reimbursements, fees, funding, or salary from an organisation that may in any way gain or lose financially from the publication of this manuscript, either now or in the future?
- Do you hold any stocks or shares in an organisation that may in any way gain or lose financially from the publication of this manuscript, either now or in the future?
- Do you hold or are you currently applying for any patents relating to the content of the manuscript?
- Have you received reimbursements, fees, funding, or salary from an organization that holds or has applied for patents relating to the content of the manuscript?
- Do you have any other financial competing interests?
- Do you have any non-financial competing interests in relation to this paper?

If you can answer no to all of the above, write 'I declare that I have no competing interests' below. If your reply is yes to any, please give details below.

I declare that I have no competing interests

I agree to the open peer review policy of the journal. I understand that my name will be included on my report to the authors and, if the manuscript is accepted for publication, my named report including any attachments I upload will be posted on the website along with the authors' responses. I agree for my report to be made available under an Open Access Creative Commons CC-BY license (http://creativecommons.org/licenses/by/4.0/). I understand that any comments which I do not wish to be included in my named report can be included as confidential comments to the editors, which will not be published.

Choose an item.

To further support our reviewers, we have joined with Publons, where you can gain additional credit to further highlight your hard work (see: https://publons.com/journal/530/gigascience). On publication of this paper, your review will be automatically added to Publons, you can then choose whether or not to claim your Publons credit. I understand this statement.

Yes Choose an item.
